# Leaf angle distribution in Johnsongrass, leaf thickness in sorghum and Johnsongrass, and association with response to *Colletotrichum sublineola*

**DOI:** 10.1038/s41598-020-79473-x

**Published:** 2020-12-18

**Authors:** Ezekiel Ahn, Gary Odvody, Louis K. Prom, Clint Magill

**Affiliations:** 1 Department of Plant Pathology and Microbiology, Texas A&M University, College Station, TX USA; 2 Texas A&M AgriLife Research, Corpus Christi, TX USA; 3 USDA-ARS Southern Plains Agricultural Research Center, College Station, TX USA

**Keywords:** Microbiology, Plant sciences

## Abstract

Basal leaf angle distribution was surveyed in twenty-one Johnsongrass cultivars near the end of the vegetative stage. The angles increased from the top to the bottom leaves, and compared to cultivated grain sorghums, the average angle was larger in Johnsongrass. When basal leaf angle distribution data were correlated with pathogenicity test data from excised-leaf assays for three isolates of *Colletotrichum sublineola*, the results showed a weak positive correlation between basal leaf angle and pathogenicity level in Johnsongrass. In order to investigate a protective role of leaf thickness to *C. sublineola*, leaf thickness was measured in three sorghum cultivars and one Johnsongrass cultivar at the 8-leaf-stage. Leaf thickness near the apex, near the base, and half-way between the two points were measured in the top four leaves of each plant. Thickness of leaf blade and midrib were recorded separately. Using an excised-leaf-assay, the three points were inoculated with *C. sublineola*, and pathogenicity level was recorded 4-days-post-inoculation. Results showed strong negative correlations between leaf midrib thickness and pathogenicity level in sorghum and Johnsongrass but not in leaf blades.

## Introduction

While sorghum (*Sorghum bicolor*) is the fifth most important cereal grown worldwide^1^, Johnsongrass (*Sorghum halepense*), a wild relative of sorghum, is considered a noxious weed^2^. Sorghum anthracnose, caused by *Colletotrichum sublineola*, is an important disease of cultivated sorghum worldwide^3^. *C. sublineola* causes anthracnose on leaf and stalk of sorghum^4^. Due to the genetic similarity between Johnsongrass and sorghum, it was hypothesized that Johnsongrass is a reservoir for *C. sublineola*, and cross-infection of *C. sublineola* was tested in a previous study between sorghum and Johnsongrass^5,6^. In those studies, 26-cultivars of Johnsongrass from different U.S. locations were inoculated at the 8-leaf-stage with three isolates of *C. sublineola* from sorghum by using an excised leaf assay^7^. Prom et al.’s excised leaf assay provides a rapid, cost effective and practical means for distinguishing resistance or susceptibility of sorghum germplasm to anthracnose, especially with a large number of sorghum germplasm^7^. Ahn et al. inoculated *C. sublineola* to Johnsongrass and confirmed that Johnsongrass can be infected with Prom et al.’s excised leaf assay^5^. Furthermore, it has been identified that midrib was more resistant to the pathogen compared to leaf blade in some sorghum and Johnsongrass lines^6^. In Johnsongrass, pathogenicity level varied among isolates and cultivars as measured on a five-point Likert scale ranging from resistant without visible fungal infection, to susceptible with formation of visible countless acervuli^6^.

Leaf angle and distribution (LAD) is an important parameter affecting the biophysical interaction of sunlight and forest canopies^8^ and in crops such as maize (*Zea mays* L.), where Genome-Wide Association Studies (GWAS) have identified several quantitative trait loci (QTL) associated with LAD^9,10^. LAD also plays a crucial role in controlling energy and mass balance in soil–vegetation–atmosphere-transfer system^11^. A study in sorghum showed that leaf angle could significantly affect biomass production^12^. In wheat (*Triticum aestivum*), a positive correlation (0.58) between leaf angle and severity to spot blotch disease, caused by *Bipolaris sorokiniana*, was found based on area under the disease progress curves (AUDPC)^13^. No attempt was made in that study to determine if the result was related to effects of leaf angle on spore retention or indirectly from energy balance. Here, basal leaf angle, which is the angle between the base of a leaf and the stem of the leaf^14^, was first surveyed in sorghum and Johnsongrass cultivars, and pathogenicity tests were conducted on plates using an excised-leaf assay^6^ which neutralizes any direct effects of erectness on inoculation efficiency.

Leaf thickness also plays an important role in leaf and plant functioning, and relates to a species' strategy of resource acquisition and use^15^. Wharton et al. showed that *C. sublineola* appressoria penetrate directly through the cuticle and epidermal cell wall of sorghum cells rather than entering via stomata^16^. Lu et al. observed that the thick cuticle of tea plants (*Camellia sinensis* (L.) O. Kuntze) impedes penetration by pathogens such as *Colletotrichum camelliae* and *C. fructicola* that cause anthracnose^17^. It is observed different levels of pathogenicity of *C. sublineola* on leaf blade versus midrib tissues in sorghum and Johnsongrass^6^. In the same study, chalcone synthase 8, a key enzyme of the flavonoid/isoflavonoid biosynthesis pathway, was highly expressed in midrib tissue of BTx623 compared to leaf blade at day 1 post-inoculation. Furthermore, it was speculated that physical characteristics such as thickness of cuticle and wax on midrib tissue compared to leaf blade tissue could be another key for different responses between midrib and leaf blade^6^. For the current study, leaf thickness on individual leaves in three sorghum cultivars (QL3, RTx2536, and Theis) and one Johnsongrass cultivar (SH1247) were measured, and response to inoculation with *C. sublineola* was also tested for potential correlation between the two factors.

## Results

### Leaf angle distribution

#### Lower leaves have higher basal leaf angles in Johnsongrass

One-way ANOVA comparing the angles based on leaf position generated p-value < 0.0001. With student’s t-test for all possible individual comparisons, leaf#7 and leaf#8 were grouped together, but others were grouped solely (Table 1). Basal leaf angles increased from the top leaves to the bottom leaves which is associated with drooping in lower leaves in Johnsongrass. In the four cultivars of sorghum, statistically meaningful difference was not found in mean basal leaf angle between leaves. These data show that at least for the 4 sorghum cultivars examined, there is less difference in basal leaf angles between leaves than for Johnsongrass.

**Table 1:** Student’s t-test for all possible individual comparisons for mean basal leaf angle distribution from leaf #1–#8 in Johnsongrass.

Leaf #	1(top)	2	3	4	5	6	7	8(bottom)
Mean ± SEM	16.70° ± 0.83	21.93° ± 0.82	25.08° ± 0.79	29.46° ± 0.9	34.98° ± 1.04	41.18° ± 1.25	46.18° ± 1.56	48.50° ± 2.09

Each leaf was solely grouped except leaf#7 and #8. Basal leaf angles were increasing from top to the bottom leaves. Similar results were generated with Tukey’s HSD test.

#### Mean basal leaf angle is greater in Johnsongrass compared to sorghum

For the cultivars examined, Johnsongrass leaves’ basal angles were greater than those of the sorghums. Specifically, based on a t-test, mean basal leaf angle measured in Johnsongrass was 32.21**°** ± 0.50 (SEM), while mean basal leaf angle in sorghum was 24.54**°** ± 1.44 (SEM) with p-value for the difference < 0.0001.

#### Mean basal leaf angle distribution statistically differed among Johnsongrass cultivars

Measured basal leaf angles (leaf #1–#8) were averaged in individual plants. Comparison of mean basal leaf angles between all twenty-one Johnsongrass cultivars and four sorghum cultivars were done by a student’s t-test for all possible group comparisons. SH1450 had the largest mean basal leaf angle with 39.23**°** ± 1.59 (SEM) which were followed by SH1002 with 36.16 ± 1.74 (SEM), SH1152 with 35.34 ± 2.38 (SEM), and SH1337 with 34.91 ± 1.84 (SEM). One-way ANOVA generated p-value < 0.0001. SH1450 has the highest mean basal leaf angle among all samples, while SH1201 has the smallest mean basal leaf angle with 23.59 ± 1.98 (SEM) among Johnsongrass samples. Three sorghum cultivars, RTx2536, QL3, and BTx623, had the smallest mean basal leaf angle among all samples [23.18 ± 2.73 (SEM), 21.94 ± 2.77 (SEM), and 18.70 ± 3.67 (SEM) respectively], while Theis showed a moderately large mean basal leaf angle [30.42 ± 2.48 (SEM)].

#### Basal leaf angle distribution is weakly correlated to Johnsongrass responses to *Colletotrichum sublineola*

Basal leaf angle was plotted versus mean pathogenicity level to three *C. sublineola* isolates that vary in virulence^5,6^. Both parametric (Pearson’s correlation) and nonparametric (Spearman’s rank correlation) tests were performed. Mean leaf angle and mean pathogenicity level to FSP2 showed weak correlations in both tests (Pearson’s correlation = 0.26 with p-value = 0.0009 and Spearman’s rank correlation = 0.16 with p-value = 0.039). Mean leaf angle and mean pathogenicity level to FSP53 is also weakly correlated in both tests (Pearson’s correlation = 0.25 with p-value = 0.0014 and Spearman’s rank correlation = 0.16 with p-value = 0.046).

### Leaf thickness

#### Characteristics of leaf thickness; blade versus midrib

Detailed information is summarized in Table 2. There is no thickness difference found between leaf blade and its midrib at the point near the apex. However, large differences were found in measurements taken near the base and half-way between the apex and base in all sorghum and Johnsongrass cultivars tested. Thickness of the leaf blade was not statistically different between the three points, but midrib thickness increased from apex to the base. Midrib thickness near the base and mid-point also increased with leaf age, that is, the midribs of the lower leaves were thicker than those of the top leaves.

**Table 2:** Leaf thickness comparisons in three sorghum and one Johnsongrass cultivars.

Cultivar	Apex		Mid-leaf		Base		Leaf to leaf comparisons	
	Leaf blade	Midrib	Leaf blade	Midrib	Leaf blade	Midrib	Leaf blade	Midrib
**QL3**								
1st leaf	0.19 ± 0.01^G^	0.19 ± 0.01^G^	0.16 ± 0.01^G^	0.44 ± 0.06^F^	0.16 ± 0.01^G^	0.78 ± 0.1^E^	0.17 ± 0.01^2^	0.47 ± 0.07^2^
2nd leaf	0.22 ± 0.01^G^	0.22 ± 0.01^G^	0.16 ± 0.01^G^	0.81 ± 0.06^E^	0.18 ± 0.01^G^	1.89 ± 0.2^C^	0.19 ± 0.01^2^	0.97 ± 0.2^1^
3rd leaf	0.20 ± 0.01^G^	0.20 ± 0.01^G^	0.16 ± 0.01^G^	0.84 ± 0.05^E^	0.21 ± 0.03^G^	2.16 ± 0.09^B^	0.19 ± 0.01^2^	1.07 ± 0.22^1^
4th leaf	0.18 ± 0.01^G^	0.18 ± 0.01^G^	0.17 ± 0.01^G^	1.03 ± 0.07^D^	0.23 ± 0.02^G^	2.49 ± 0.08^A^	0.19 ± 0.01^2^	1.24 ± 0.26^1^
Leaf position average	0.20 ± 0.01*	0.20 ± 0.01*	0.16 ± 0.01*	0.78 ± 0.06**	0.19 ± 0.01*	1.83 ± 0.16***		
**RTx2536**								
1st leaf	0.27 ± 0.01^EF^	0.27 ± 0.01^EF^	0.18 ± 0.01^F^	0.45 ± 0.02^E^	0.21 ± 0.01^EF^	1.38 ± 0.12^C^	0.22 ± 0.02^3^	0.70 ± 0.18^2,3^
2nd leaf	0.24 ± 0.03^EF^	0.24 ± 0.03^EF^	0.18 ± 0.01^F^	1.06 ± 0.08^D^	0.21 ± 0.01^EF^	2.55 ± 0.18^B^	0.21 ± 0.01^3^	1.29 ± 0.34^1,2^
3rd leaf	0.22 ± 0.01^EF^	0.22 ± 0.01^EF^	0.19 ± 0.01^EF^	1.34 ± 0.06^C^	0.2 ± 0.02^EF^	3.18 ± 0.34^A^	0.20 ± 0.01^3^	1.58 ± 0.44^1^
4th leaf	0.20 ± 0.01^EF^	0.20 ± 0.01^EF^	0.19 ± 0.01^F^	1.39 ± 0.12^C^	0.21 ± 0.01^EF^	2.99 ± 0.12^A^	0.20 ± 0.01^3^	1.53 ± 0.41^1^
Leaf position average	0.23 ± 0.01***	0.23 ± 0.01***	0.19 ± 0.01***	1.06 ± 0.1**	0.21 ± 0.01***	2.53 ± 0.23*		
**Theis**								
1st leaf	0.19 ± 0.02^FG^	0.19 ± 0.02^FG^	0.15 ± 0.01^G^	0.35 ± 0.02^F^	0.18 ± 0.02^G^	0.76 ± 0.08^E^	0.17 ± 0.01^4^	0.43 ± 0.08^3,4^
2nd leaf	0.14 ± 0.01^G^	0.14 ± 0.01^G^	0.16 ± 0.01^G^	0.68 ± 0.03^E^	0.21 ± 0.03^FG^	1.66 ± 0.11^C^	0.17 ± 0.01^4^	0.83 ± 0.19^2,3^
3rd leaf	0.20 ± 0.01^FG^	0.20 ± 0.01^FG^	0.16 ± 0.01^G^	0.81 ± 0.06^E^	0.17 ± 0.01^G^	1.92 ± 0.9^B^	0.18 ± 0.01^4^	0.98 ± 0.22^1,2^
4th leaf	0.22 ± 0.05^FG^	0.22 ± 0.05^FG^	0.17 ± 0.01^G^	1.27 ± 0.05^D^	0.23 ± 0.01^FG^	2.48 ± 0.21^A^	0.21 ± 0.02^4^	1.32 ± 0.28^1^
Leaf position average	0.19 ± 0.01***	0.19 ± 0.01***	0.16 ± 0.01***	0.78 ± 0.09**	0.20 ± 0.04***	1.70 ± 0.17*		
**SH1247**								
1st leaf	0.25 ± 0.01^DE^	0.25 ± 0.01^DE^	0.23 ± 0.03^E^	0.54 ± 0.06^CD^	0.21 ± 0.02^E^	1.27 ± 0.19^B^	0.23 ± 0.01^2^	0.69 ± 0.13^1^
2nd leaf	0.22 ± 0.01^E^	0.22 ± 0.01^E^	0.19 ± 0.01^E^	0.67 ± 0.07^C^	0.21 ± 0.02^E^	2.02 ± 0.24^A^	0.21 ± 0.01^2^	0.97 ± 0.22^1^
3rd leaf	0.24 ± 0.02^E^	0.24 ± 0.02^E^	0.17 ± 0.01^E^	0.78 ± 0.07^C^	0.22 ± 0.02^E^	2.14 ± 0.22^A^	0.21 ± 0.01^2^	1.05 ± 0.22^1^
4th leaf	0.22 ± 0.01^E^	0.22 ± 0.01^E^	0.17 ± 0.01^E^	0.78 ± 0.10^C^	0.19 ± 0.01^E^	2.09 ± 0.29^A^	0.19 ± 0.01^2^	1.03 ± 0.23^1^
Leaf position average	0.23 ± 0.01***	0.23 ± 0.01***	0.19 ± 0.01***	0.69 ± 0.04**	0.20 ± 0.01***	1.88 ± 0.14*		

A student’s t-test for all possible group comparisons within each cultivar for leaf thickness at three points (near apex, near base, and half-way between apex and base) as were the leaf to leaf comparisons and leaf position comparisons. Upper to lower leaves were labeled as 1st leaf to 4th leaf. Leaf blade and midrib tissues were measured separately. Unit = mm. Statistically different groups are labeled with letters for specific locations, number(s) for different leaves, and number of asterisk marks for leaf blade/midrib at apex/middle (half-way)/base.

#### Midrib/leaf blade and midrib combined thickness has strong negative correlation to sorghum and Johnsongrass responses to *Colletotrichum sublineola*

Based on leaf thickness measurement and the excised leaf assay results, leaf thickness and pathogenicity level of leaf blade and midrib thickness are identical near the leaf tip, so in order to remove redundancy for Spearman’s rank correlation test of the leaf blade and midrib combination, only one value was used for each. For each type of tissue alone and pathogenicity level correlation tests, no data were omitted. However, even including those same values twice had very little effect on Spearman’s rank correlation. Spearman’s correlation test revealed a strong negative correlation between midrib thickness/leaf blade and midrib combined thickness and pathogenicity levels in all four cultivars tested (Table 3). In every case, the results show clear negative correlations between midrib thickness and pathogenicity level.

**Table 3:** Spearman’s rank correlation between leaf blade, midrib, leaf blade and midrib combined, and pathogenicity level.

Cultivar	QL3	RTx2536	Theis	SH1247
**Leaf blade-pathogenicity level**				
Correlation	0.15	0.09	0.14	− 0.12
p-value	0.26	0.59	0.33	0.37
**Midrib-pathogenicity level**				
Correlation	− 0.73	− 0.62	− 0.61	− 0.76
p-value	< 0.0001	< 0.0001	< 0.0001	< 0.0001
**Leaf blade and midrib-pathogenicity level**				
Correlation	− 0.73	− 0.72	− 0.54	− 0.70
p-value	< 0.0001	< 0.0001	< 0.0001	< 0.0001

Midrib thickness is negatively correlated with pathogenicity level.

## Discussion

The angle of a leaf’s surface to the horizontal directly determines the flux of solar radiation per unit leaf area^18^. Hence, steeper leaf angles increase light capture when the sun is at low angles in the sky (morning/afternoon and winter), while decreasing light capture from higher angles (midday and summer)^18^. The benefits of steeper leaf angles include a reduction in midday head-loads, a decrease in the susceptibility to mild or severe photoinhibition, and minimizing water-use with respect to daily carbon gain^18^. In maize, plant architecture is a key factor for high productivity because ideal plant architecture with an erect leaf angle and optimum leaf orientation value allow for more efficient light capture during photosynthesis and better wind circulation under dense planting conditions^9^, and sorghum plant structure is very similar to that of maize. In a plant pathology perspective, increased leaf angle was associated with droopy leaves and increased severity to spot blotch (*Bipolaris sorokiniana*) disease in wheat, with a correlation of 0.58^13^. While that study involved inoculation of plot-grown plants, here the host plant was Johnsongrass tested with the known pathogen *C. sublineola* using an excised-leaf assy. The excised-assay uses excised leaves placed flat on half strength PDA media for *C. sublineola* inoculation, and any effect of leaf erectness such as reduced spore access is eliminated. Based on the results, even after neutralizing the effect of leaf erectness, a weakly positive correlation between leaf angle and Johnsongrass responses to *C. sublineola* isolates was detected for 2 of 3 races of the pathogen. While the genotypes with different degrees of leaf erectness trait could also differ in speed or level of host defense response, irrespective of leaf angle, an attractive alternative is that energy availability for defense response metabolism prior to inoculation as a result of more erect leaves may be the more important factor. It is reported that even though the Johnsongrass cultivars showed signs of induced defense response following greenhouse spray inoculations with FSP53, pathogen reproduction was never successful in terms of appressorium production with one exception, but differential responses were detected using an excised leaf assay^5^. FSP35 infected all twenty-six cultivars tested with high pathogenicity scores with little variability, resulting in no correlation for FSP35 versus pathogenicity level^5,6^.

Conversely, Spearman’s correlation test for leaf midrib thickness and pathogenicity level revealed a strong negative correlation of nearly − 0.70 in most cases. Leaf blade tissue maintains nearly the same thickness through the length of the leaf, whereas the midrib increases thickness across the leaf. Physical structures provide protection against desiccation and external environmental stresses in plants^19^. However, it has been demonstrated that cuticle thickness in nine feral *Phlox* taxa, including *Phlox carolina* L., *P. drummondii* ssp. *mcallisteri* (Whitehouse) Wherry, and *P. Pilosa* ssp. *detonsa* (Gray) Wherry, is not correlated with resistance to a powdery mildew causing pathogen, *Erysiphe cichoracearum*^20^ and as mentioned above, *C. sublineola* appressoria penetrate directly through the cuticle and epidermal cell wall of sorghum cells rather than entering via stomata^16^. Most information regarding the composition of midrib tissue in sorghum involves the effects of two different mutations that alter the lignin present leading to a brown midrib phenotype as a result of altered lignin composition that also improves digestibility by animals and ethanol production^21^. The midribs of members of the grass family such as sorghum are highly lignified which provides a means of leaf support and cross-linking of lignin to polysaccharides adds rigidity to the cell and strengthens the cell wall^22^. In addition, lignin synthesis can be rapidly activated in host defense versus pathogens as part of the phenylalanine ammonia lyase pathway that leads to production of hydroxycinnamyl alcohols, the precursors of lignin^21^. It has been suggested that pathway intermediates that accumulate may be more toxic to fungi than the deterrent effect of increased lignin^23^. As indicated previously, at least one step in the PAL pathway was shown to be induced earlier in midrib than leaf blade after inoculation with *C. sublineola* spores. Thus, increased resistance in the thicker midribs may be a consequence of increased lignification combined with active defense.

## Methods

### Plant material preparation

Leaf angle distribution. Twenty-one cultivars of Johnsongrass (see Supplementary Table S1) were provided by Jacob Barney (Virginia Tech University) in the form of rhizomes that were transplanted into 2-gallon plastic round pots filled with Sungro professional growing mix and grown in a greenhouse in College Station, TX, USA. Four sorghum cultivars, BTx623, QL3, RTx2536, and Theis, were grown as a comparison between sorghum and Johnsongrass cultivars. Water and additional nutritional supplements were provided regularly. At booting stage (end of vegetative state), most plants generated 6–8 leaves with a few exceptions where only 5 leaves emerged. Leaves were numbered from top to the bottom as 1 through 8.

Leaf thickness. Seed from three sorghum cultivars (QL3, RTx2536, and Theis) were planted in 2-gallon plastic round pots filled with Sungro professional growing mix and grown in a greenhouse in College Station, TX, USA. SH1247, one of the cultivars from the leaf angle distribution study, was planted in the form of rhizomes in 2-gallon plastic round pots from Nursery supplies INC filled with Sungro professional growing mix. At the 8-leaf-stage, the top four leaves (labeled as #1 through #4) were collected to measure leaf thickness.

### Measurements

Leaf angle distribution. At booting stage, basal leaf angles, the adaxial angle between a leaf and a stem, were measured with a protractor^14^. At least three individuals were measured for each cultivar. In sum, 1326 basal leaf angles from 162 individuals in 21 Johnsongrass cultivars and 19 individuals in 4 sorghum cultivars were measured and recorded (see Supplementary Table S1).

Leaf thickness. Leaf thickness near the apex, near the base, and half-way between the two points were measured in the top four leaves by using a Mitutoyo 547-500S Digimatic Digital Thickness Gage. Thickness of leaf blade and midrib were recorded separately (see Supplementary Table S2). Many lower leaves of the plants grown under the greenhouse condition lost their turgidity, so thicknesses were measured only on the top four leaves.

### Excised-leaf spot inoculation

Disease ratings for the 21 Johnsongrass cultivars used in the leaf angle study were available from a prior study^6^. In that study, 26-cultivars of Johnsongrass from different U.S. locations were inoculated near the end of vegetative stage with three isolates of *C. sublineola* from sorghum by using the excised leaf assay as described by Prom et al.^7^. Three to four leaf segments were detached and evaluated for *C. sublineola* inoculation scoring from each plant, and three different individual plants were used for each cultivar. Mean pathogenicity levels on a scale of 1–5 were provided for each of the Johnsongrass cultivars.

The same excised leaf assay was used to inoculate QL3, RTx2536, Theis, and SH1247 in the leaf thickness experiments. A mixture of three virulent strains of *C. sublineola* (FSP 2, FSP 35, FSP 53) isolated from sorghum was used to determine host response. Plants were inoculated at pre-marked points of leaf blade and midrib tissues at near the apex, near the base, and half-way between the two points. For preparation of inoculum, *C. sublineola* was inoculated onto half strength potato dextrose agar (PDA) medium and grown at room temperature for 10–14 days. Approximately 1–2 ml of sterile water was added to each plate, and a sterile spatula was used to scrape and remove colony growth of *C. sublineola* from the plate. This suspension was filtered through four layers of cheesecloth to remove mycelium then before the spores were diluted to a final concentration of ~ 1 × 10^6^ conidia/ml. Excised leaf pieces, placed adaxial side up on the agar surface, were inoculated on each side of the leaf blade and one spot on the midrib using 5 μl of the spore suspension. Excised leaves were observed under an Olympus BX60 microscope at 96 h post-inoculation. Susceptibility was scored using a 1–10 scale: (1) fully resistant without visible fungal infection, (2) fungal germ tube formed, (3) fungal bed formed with some imperfectly formed acervuli, (4) 1–5 acervuli perfectly formed, (5) 6–10 acervuli perfectly formed, (6) 11–15 acervuli perfectly formed, (7) 16–20 acervuli perfectly formed, (8) 21–25 acervuli perfectly formed, (9) 26–30 acervuli perfectly formed, and (10) 31 or more acervuli perfectly formed. The experiment was repeated three to five times with fresh sets of plants.

### Statistical analysis

Leaf angle distribution. A student’s t-test for all possible group comparisons was performed with JMP Pro 14 to compare basal leaf angles between leaves 1–8 in 21 Johnsongrass and 4 sorghum cultivars. One-way ANOVA was used to compare basal leaf angles based on leaf position and cultivars. The mean pathogenicity levels of 21 cultivars inoculated separately with spores of *C. sublineola* isolates FSP2, FSP35, and FSP53 from the previous study^6^ were tested for correlation with newly collected basal leaf angle distribution data. Basal leaf angle is a continuous variable. Mean pathogenicity levels of each cultivar were generated based on de Winter and Dodou’s concept of treating Likert scale data as a continuous variable^24^. Here two approaches were made: (1) Mean pathogenicity was treated as a continuous variable, and Pearson’s correlation was tested between mean pathogenicity of each cultivar and basal leaf angle distribution data, and (2) Mean pathogenicity was treated as ordinal data, and Spearman’s rank correlation was performed.

Leaf thickness. A student’s t-test for all possible group comparisons was performed with JMP Pro 14 to compare leaf thickness between the three points and between leaf and midrib in three sorghum and one Johnsongrass cultivars. The collected leaf thickness data was combined with the results from an excised leaf infection assay and Spearman’s rank correlation test was performed.

## Supplementary Information

Supplementary Table S1.

Supplementary Table S2.
