# Author Correction: Leaf angle distribution in Johnsongrass, leaf thickness in sorghum and Johnsongrass, and association with response to *Colletotrichum sublineola*

**DOI:** 10.1038/s41598-021-89272-7

**Published:** 2021-05-07

**Authors:** Ezekiel Ahn, Gary Odvody, Louis K. Prom, Clint Magill

**Affiliations:** 1 Department of Plant Pathology and Microbiology, Texas A&M University, College Station, TX USA; 2 Texas A&M AgriLife Research, Corpus Christi, TX USA; 3 USDA-ARS Southern Plains Agricultural Research Center, College Station, TX USA

Correction to: *Scientific Reports* https://doi.org/10.1038/s41598-020–9473-x, published online 18 December 2020

The original version of this Article contained errors.

Table 2 contained several errors in the values given for the “Mid-leaf”, “Midrib” and “Base”, “Leaf blade” for “1st leaf”, “2nd leaf”, “3rd leaf” and “4th leaf” for Cultivar RTx2536.

In addition, there were four instances where the standard deviation calculation instead of standard error calculation was erroneously used.

The original Table 2 and accompanying legend appear below.

**Table 2:** Leaf thickness comparisons in three sorghum and one Johnsongrass cultivars.

Cultivar	Apex		Mid-leaf		Base		Leaf to leaf comparisons	
	Leaf blade	Midrib	Leaf blade	Midrib	Leaf blade	Midrib	Leaf blade	Midrib
**QL3**								
1st leaf	0.19 ± 0.01^G^	0.19 ± 0.01^G^	0.16 ± 0.01^G^	0.44 ± 0.06^F^	0.16 ± 0.01^G^	0.78 ± 0.1^E^	0.17 ± 0.01^2^	0.47 ± 0.07^2^
2nd leaf	0.22 ± 0.01^G^	0.22 ± 0.01^G^	0.16 ± 0.01^G^	0.81 ± 0.06^E^	0.18 ± 0.01^G^	1.89 ± 0.2^C^	0.19 ± 0.01^2^	0.97 ± 0.2^1^
3rd leaf	0.20 ± 0.01^G^	0.20 ± 0.01^G^	0.16 ± 0.01^G^	0.84 ± 0.05^E^	0.21 ± 0.03^G^	2.16 ± 0.09^B^	0.19 ± 0.01^2^	1.07 ± 0.22^1^
4th leaf	0.18 ± 0.01^G^	0.18 ± 0.01^G^	0.17 ± 0.02^G^	1.03 ± 0.07^D^	0.23 ± 0.02^G^	2.49 ± 0.08^A^	0.19 ± 0.01^2^	1.24 ± 0.26^1^
Leaf position average	0.20 ± 0.01*	0.20 ± 0.01*	0.16 ± 0.01*	0.78 ± 0.06**	0.19 ± 0.01*	1.83 ± 0.16***		
**RTx2536**								
1st leaf	0.27 ± 0.01^EF^	0.27 ± 0.01^EF^	0.18 ± 0.01^F^	1.38 ± 0.02^E^	0.21 ± 0.01^EF^	1.38 ± 0.12^C^	0.22 ± 0.02^3^	0.70 ± 0.18^2,3^
2nd leaf	0.24 ± 0.03^EF^	0.24 ± 0.03^EF^	0.18 ± 0.01^F^	2.55 ± 0.08^D^	0.21 ± 0.01^EF^	2.55 ± 0.18^B^	0.21 ± 0.01^3^	1.29 ± 0.34^1,2^
3rd leaf	0.22 ± 0.01^EF^	0.22 ± 0.01^EF^	0.19 ± 0.01^EF^	3.18 ± 0.06^C^	0.2 ± 0.02^EF^	3.18 ± 0.34^A^	0.20 ± 0.01^3^	1.58 ± 0.44^1^
4th leaf	0.20 ± 0.01^EF^	0.20 ± 0.01^EF^	0.19 ± 0.01^F^	2.99 ± 0.12^C^	0.21 ± 0.01^EF^	2.99 ± 0.12^A^	0.20 ± 0.01^3^	1.53 ± 0.41^1^
Leaf position average	0.23 ± 0.01***	0.23 ± 0.04***	0.19 ± 0.01***	1.06 ± 0.41**	0.21 ± 0.01***	2.53 ± 0.79*		
**Theis**								
1st leaf	0.19 ± 0.02^FG^	0.19 ± 0.02^FG^	0.15 ± 0.01^G^	0.35 ± 0.02^F^	0.18 ± 0.02^G^	0.76 ± 0.08^E^	0.17 ± 0.01^4^	0.43 ± 0.08^3,4^
2nd leaf	0.14 ± 0.01^G^	0.14 ± 0.01^G^	0.16 ± 0.01^G^	0.68 ± 0.03^E^	0.21 ± 0.03^FG^	1.66 ± 0.11^C^	0.17 ± 0.01^4^	0.83 ± 0.19^2,3^
3rd leaf	0.20 ± 0.01^FG^	0.20 ± 0.01^FG^	0.16 ± 0.01^G^	0.81 ± 0.06^E^	0.17 ± 0.01^G^	1.92 ± 0.9^B^	0.18 ± 0.01^4^	0.98 ± 0.22^1,2^
4th leaf	0.22 ± 0.05^FG^	0.22 ± 0.05^FG^	0.17 ± 0.01^G^	1.27 ± 0.05^D^	0.23 ± 0.01^FG^	2.48 ± 0.21^A^	0.21 ± 0.02^4^	1.32 ± 0.28^1^
Leaf position average	0.19 ± 0.01***	0.19 ± 0.01***	0.16 ± 0.01***	0.78 ± 0.09**	0.20 ± 0.04***	1.70 ± 0.17*		
**SH1247**								
1st leaf	0.25 ± 0.01^DE^	0.25 ± 0.01^DE^	0.23 ± 0.03^E^	0.54 ± 0.06^CD^	0.21 ± 0.02^E^	1.27 ± 0.19^B^	0.23 ± 0.01^2^	0.69 ± 0.13^1^
2nd leaf	0.22 ± 0.01^E^	0.22 ± 0.01^E^	0.19 ± 0.01^E^	0.67 ± 0.07^C^	0.21 ± 0.02^E^	2.02 ± 0.24^A^	0.21 ± 0.01^2^	0.97 ± 0.22^1^
3rd leaf	0.24 ± 0.02^E^	0.24 ± 0.02^E^	0.17 ± 0.01^E^	0.78 ± 0.07^C^	0.22 ± 0.02^E^	2.14 ± 0.22^A^	0.21 ± 0.01^2^	1.05 ± 0.22^1^
4th leaf	0.22 ± 0.01^E^	0.22 ± 0.01^E^	0.17 ± 0.01^E^	0.78 ± 0.10^C^	0.19 ± 0.01^E^	2.09 ± 0.29^A^	0.19 ± 0.01^2^	1.03 ± 0.23^1^
Leaf position average	0.23 ± 0.01***	0.23 ± 0.01***	0.19 ± 0.01***	0.69 ± 0.04**	0.20 ± 0.01***	1.88 ± 0.14*		

A student’s t-test for all possible group comparisons within each cultivar for leaf thickness at three points (near apex, near base, and half-way between apex and base) as were the leaf to leaf comparisons and leaf position comparisons. Upper to lower leaves were labeled as 1st leaf to 4th leaf. Leaf blade and midrib tissues were measured separately. Unit = mm. Statistically different groups are labeled with letters for specific locations, number(s) for different leaves, and number of asterisk marks for leaf blade/midrib at apex/middle (half-way)/base.

Furthermore, in the Discussion section, reference 13 was omitted.

“In a plant pathology perspective, increased leaf angle was associated with droopy leaves and increased severity to spot blotch (*Bipolaris sorokiniana*) disease in wheat, with a correlation of 0.58 (Joshi A and Chand R 2002)”

now reads:

“In a plant pathology perspective, increased leaf angle was associated with droopy leaves and increased severity to spot blotch (*Bipolaris sorokiniana*) disease in wheat, with a correlation of 0.58^13^”

The original Article has been corrected.

