# Supplementary material for: Leaf angle distribution in Johnsongrass, leaf thickness in sorghum and Johnsongrass, and association with response to Colletotrichum sublineola
Source: Sci Rep. 2020 Dec 18;10:22320. doi: 10.1038/s41598-020-79473-x (PMC7749152; doi:10.1038/s41598-020-79473-x)
Supplement: Supplementary file 1 — Supplementary Table S1. [file 41598_2020_79473_MOESM1_ESM.docx]

Title of the manuscript: Leaf angle distribution in Johnsongrass, leaf thickness in sorghum and Johnsongrass, and association with response to *Colletotrichum sublineola*

Full author list: EZEKIEL AHN^1^, GARY ODVODY^2^, LOUIS K. PROM^3^ and CLINT MAGILL^1^

^1^*Department of Plant Pathology & Microbiology, Texas A&M University, College Station, TX, USA*

^2^ *Texas A&M AgriLife Research, Corpus Christi, TX, USA*

^3^ *USDA-ARS Southern Plains Agricultural Research Center, College Station, TX, USA*

Correspondence to: C. Magill. E-mail: c-magill@tamu.edu

Supplementary Table S1. Raw data of leaf angle. Name of plant cultivars, leaf angle, leaf number, plant, collected state and home habitat information are listed.

| Cultivar | Leaf angle (°) | Leaf | Sorghum(S)  or Johnsongrass(J) | State | Home habitat |
| --- | --- | --- | --- | --- | --- |
| BTx623 | 10 | 1 | S |  |  |
| BTx623 | 15.7 | 2 | S |  |  |
| BTx623 | 14.8 | 3 | S |  |  |
| BTx623 | 17 | 4 | S |  |  |
| BTx623 | 15.5 | 5 | S |  |  |
| BTx623 | 25.3 | 6 | S |  |  |
| BTx623 | 17.9 | 7 | S |  |  |
| BTx623 | 23 | 8 | S |  |  |
| BTx623 | 13.4 | 1 | S |  |  |
| BTx623 | 14.5 | 2 | S |  |  |
| BTx623 | 15.5 | 3 | S |  |  |
| BTx623 | 30.7 | 4 | S |  |  |
| BTx623 | 18.3 | 5 | S |  |  |
| BTx623 | 24.5 | 6 | S |  |  |
| BTx623 | 21.5 | 7 | S |  |  |
| BTx623 | 13.6 | 1 | S |  |  |
| BTx623 | 17 | 2 | S |  |  |
| BTx623 | 26.5 | 3 | S |  |  |
| BTx623 | 14.1 | 4 | S |  |  |
| BTx623 | 23.3 | 5 | S |  |  |
| BTx623 | 20.6 | 6 | S |  |  |
| QL3 | 23.5 | 1 | S |  |  |
| QL3 | 27.1 | 2 | S |  |  |
| QL3 | 17.6 | 3 | S |  |  |
| QL3 | 18.7 | 4 | S |  |  |
| QL3 | 17.1 | 5 | S |  |  |
| QL3 | 21.1 | 6 | S |  |  |
| QL3 | 22.7 | 7 | S |  |  |
| QL3 | 55.1 | 1 | S |  |  |
| QL3 | 55.1 | 2 | S |  |  |
| QL3 | 32.8 | 3 | S |  |  |
| QL3 | 27.3 | 4 | S |  |  |
| QL3 | 22.2 | 5 | S |  |  |
| QL3 | 15.2 | 6 | S |  |  |
| QL3 | 15.2 | 7 | S |  |  |
| QL3 | 23.2 | 1 | S |  |  |
| QL3 | 23.1 | 2 | S |  |  |
| QL3 | 23.1 | 3 | S |  |  |
| QL3 | 26 | 4 | S |  |  |
| QL3 | 13.2 | 5 | S |  |  |
| QL3 | 18.3 | 6 | S |  |  |
| QL3 | 19.4 | 7 | S |  |  |
| QL3 | 14.8 | 1 | S |  |  |
| QL3 | 16 | 2 | S |  |  |
| QL3 | 18 | 3 | S |  |  |
| QL3 | 18 | 4 | S |  |  |
| QL3 | 16.1 | 5 | S |  |  |
| QL3 | 19.7 | 6 | S |  |  |
| QL3 | 28.2 | 7 | S |  |  |
| QL3 | 20.6 | 8 | S |  |  |
| QL3 | 13.5 | 1 | S |  |  |
| QL3 | 15.1 | 2 | S |  |  |
| QL3 | 15.7 | 3 | S |  |  |
| QL3 | 14.8 | 4 | S |  |  |
| QL3 | 14.8 | 5 | S |  |  |
| QL3 | 18.3 | 6 | S |  |  |
| QL3 | 25.5 | 7 | S |  |  |
| QL3 | 25.5 | 8 | S |  |  |
| RTx2536 | 30.2 | 1 | S |  |  |
| RTx2536 | 30.8 | 2 | S |  |  |
| RTx2536 | 25.7 | 3 | S |  |  |
| RTx2536 | 25.7 | 4 | S |  |  |
| RTx2536 | 22.2 | 5 | S |  |  |
| RTx2536 | 23.3 | 6 | S |  |  |
| RTx2536 | 22.5 | 7 | S |  |  |
| RTx2536 | 16.9 | 1 | S |  |  |
| RTx2536 | 17.4 | 2 | S |  |  |
| RTx2536 | 18.8 | 3 | S |  |  |
| RTx2536 | 13.6 | 4 | S |  |  |
| RTx2536 | 22.8 | 5 | S |  |  |
| RTx2536 | 24.4 | 6 | S |  |  |
| RTx2536 | 12.2 | 7 | S |  |  |
| RTx2536 | 12 | 1 | S |  |  |
| RTx2536 | 13.5 | 2 | S |  |  |
| RTx2536 | 16.4 | 3 | S |  |  |
| RTx2536 | 19.3 | 4 | S |  |  |
| RTx2536 | 18.1 | 5 | S |  |  |
| RTx2536 | 21 | 6 | S |  |  |
| RTx2536 | 24.3 | 7 | S |  |  |
| RTx2536 | 26.9 | 8 | S |  |  |
| RTx2536 | 13.6 | 1 | S |  |  |
| RTx2536 | 17.8 | 2 | S |  |  |
| RTx2536 | 21.6 | 3 | S |  |  |
| RTx2536 | 19.6 | 4 | S |  |  |
| RTx2536 | 20.8 | 5 | S |  |  |
| RTx2536 | 25.3 | 6 | S |  |  |
| RTx2536 | 34.4 | 7 | S |  |  |
| RTx2536 | 27.2 | 8 | S |  |  |
| RTx2536 | 14.2 | 1 | S |  |  |
| RTx2536 | 19.4 | 2 | S |  |  |
| RTx2536 | 26.6 | 3 | S |  |  |
| RTx2536 | 38.9 | 4 | S |  |  |
| RTx2536 | 53.4 | 5 | S |  |  |
| RTx2536 | 32.7 | 6 | S |  |  |
| RTx2536 | 29.4 | 7 | S |  |  |
| RTx2536 | 27.9 | 8 | S |  |  |
| SH1002 | 14.1 | 1 | J | VA |  |
| SH1002 | 20 | 2 | J | VA |  |
| SH1002 | 33.6 | 3 | J | VA |  |
| SH1002 | 41.6 | 4 | J | VA |  |
| SH1002 | 61.9 | 5 | J | VA |  |
| SH1002 | 61.9 | 6 | J | VA |  |
| SH1002 | 85.2 | 7 | J | VA |  |
| SH1002 | 16.8 | 1 | J | VA |  |
| SH1002 | 20.6 | 2 | J | VA |  |
| SH1002 | 31.4 | 3 | J | VA |  |
| SH1002 | 29.2 | 4 | J | VA |  |
| SH1002 | 44.5 | 5 | J | VA |  |
| SH1002 | 35.5 | 6 | J | VA |  |
| SH1002 | 67.2 | 7 | J | VA |  |
| SH1002 | 15.3 | 1 | J | VA |  |
| SH1002 | 17.7 | 2 | J | VA |  |
| SH1002 | 16.2 | 3 | J | VA |  |
| SH1002 | 51.9 | 4 | J | VA |  |
| SH1002 | 34.3 | 5 | J | VA |  |
| SH1002 | 72.1 | 6 | J | VA |  |
| SH1002 | 15.1 | 1 | J | VA |  |
| SH1002 | 16.7 | 2 | J | VA |  |
| SH1002 | 21.8 | 3 | J | VA |  |
| SH1002 | 21.4 | 4 | J | VA |  |
| SH1002 | 38.3 | 5 | J | VA |  |
| SH1002 | 56.6 | 6 | J | VA |  |
| SH1002 | 12.7 | 1 | J | VA |  |
| SH1002 | 29 | 2 | J | VA |  |
| SH1002 | 22.7 | 3 | J | VA |  |
| SH1002 | 32 | 4 | J | VA |  |
| SH1002 | 36.3 | 5 | J | VA |  |
| SH1002 | 77.6 | 6 | J | VA |  |
| SH1002 | 72.3 | 7 | J | VA |  |
| SH1002 | 14.1 | 1 | J | VA |  |
| SH1002 | 18.3 | 2 | J | VA |  |
| SH1002 | 26.9 | 3 | J | VA |  |
| SH1002 | 33.8 | 4 | J | VA |  |
| SH1002 | 38.3 | 5 | J | VA |  |
| SH1002 | 41.9 | 6 | J | VA |  |
| SH1002 | 82.3 | 7 | J | VA |  |
| SH1002 | 12.9 | 1 | J | VA |  |
| SH1002 | 17.1 | 2 | J | VA |  |
| SH1002 | 20.9 | 3 | J | VA |  |
| SH1002 | 21.6 | 4 | J | VA |  |
| SH1002 | 35.5 | 5 | J | VA |  |
| SH1002 | 37.5 | 6 | J | VA |  |
| SH1002 | 62.8 | 7 | J | VA |  |
| SH1002 | 55.8 | 8 | J | VA |  |
| SH1002 | 9.8 | 1 | J | VA |  |
| SH1002 | 14.8 | 2 | J | VA |  |
| SH1002 | 20.2 | 3 | J | VA |  |
| SH1002 | 21.7 | 4 | J | VA |  |
| SH1002 | 35.6 | 5 | J | VA |  |
| SH1002 | 36.6 | 6 | J | VA |  |
| SH1002 | 47.1 | 7 | J | VA |  |
| SH1002 | 45 | 8 | J | VA |  |
| SH1002 | 12.8 | 1 | J | VA |  |
| SH1002 | 16 | 2 | J | VA |  |
| SH1002 | 24.4 | 3 | J | VA |  |
| SH1002 | 29.1 | 4 | J | VA |  |
| SH1002 | 48.4 | 5 | J | VA |  |
| SH1002 | 64.8 | 6 | J | VA |  |
| SH1002 | 68.9 | 7 | J | VA |  |
| SH1002 | 11.1 | 1 | J | VA |  |
| SH1002 | 12.4 | 2 | J | VA |  |
| SH1002 | 20.5 | 3 | J | VA |  |
| SH1002 | 39.7 | 4 | J | VA |  |
| SH1002 | 31.3 | 5 | J | VA |  |
| SH1002 | 46.5 | 6 | J | VA |  |
| SH1002 | 58.9 | 7 | J | VA |  |
| SH1002 | 64.5 | 8 | J | VA |  |
| SH1002 | 13 | 1 | J | VA |  |
| SH1002 | 26.1 | 2 | J | VA |  |
| SH1002 | 26.2 | 3 | J | VA |  |
| SH1002 | 29.3 | 4 | J | VA |  |
| SH1002 | 35 | 5 | J | VA |  |
| SH1002 | 49.8 | 6 | J | VA |  |
| SH1002 | 49.8 | 7 | J | VA |  |
| SH1002 | 50 | 8 | J | VA |  |
| SH1002 | 10 | 1 | J | VA |  |
| SH1002 | 18.6 | 2 | J | VA |  |
| SH1002 | 26.1 | 3 | J | VA |  |
| SH1002 | 37.1 | 4 | J | VA |  |
| SH1002 | 32.9 | 5 | J | VA |  |
| SH1002 | 50.3 | 6 | J | VA |  |
| SH1002 | 62.3 | 7 | J | VA |  |
| SH1002 | 68.7 | 8 | J | VA |  |
| SH1002 | 9.8 | 1 | J | VA |  |
| SH1002 | 26.2 | 2 | J | VA |  |
| SH1002 | 28.8 | 3 | J | VA |  |
| SH1002 | 36.3 | 4 | J | VA |  |
| SH1002 | 54.9 | 5 | J | VA |  |
| SH1002 | 65.9 | 6 | J | VA |  |
| SH1002 | 68.9 | 7 | J | VA |  |
| SH1048 | 12.1 | 1 | J | TX | Agricultural |
| SH1048 | 13.2 | 2 | J | TX | Agricultural |
| SH1048 | 16.4 | 3 | J | TX | Agricultural |
| SH1048 | 25.8 | 4 | J | TX | Agricultural |
| SH1048 | 47.2 | 5 | J | TX | Agricultural |
| SH1048 | 62.2 | 6 | J | TX | Agricultural |
| SH1048 | 11.9 | 1 | J | TX | Agricultural |
| SH1048 | 14.3 | 2 | J | TX | Agricultural |
| SH1048 | 14.8 | 3 | J | TX | Agricultural |
| SH1048 | 25.8 | 4 | J | TX | Agricultural |
| SH1048 | 35.5 | 5 | J | TX | Agricultural |
| SH1048 | 49.5 | 6 | J | TX | Agricultural |
| SH1048 | 50.9 | 7 | J | TX | Agricultural |
| SH1048 | 11.9 | 1 | J | TX | Agricultural |
| SH1048 | 13.9 | 2 | J | TX | Agricultural |
| SH1048 | 14.8 | 3 | J | TX | Agricultural |
| SH1048 | 19.9 | 4 | J | TX | Agricultural |
| SH1048 | 29.2 | 5 | J | TX | Agricultural |
| SH1048 | 36.8 | 6 | J | TX | Agricultural |
| SH1048 | 44 | 7 | J | TX | Agricultural |
| SH1048 | 10.9 | 1 | J | TX | Agricultural |
| SH1048 | 14.1 | 2 | J | TX | Agricultural |
| SH1048 | 13.8 | 3 | J | TX | Agricultural |
| SH1048 | 26.4 | 4 | J | TX | Agricultural |
| SH1048 | 32.4 | 5 | J | TX | Agricultural |
| SH1048 | 39.9 | 6 | J | TX | Agricultural |
| SH1048 | 51.3 | 7 | J | TX | Agricultural |
| SH1094 | 14.1 | 1 | J | TX | Agricultural |
| SH1094 | 14.5 | 2 | J | TX | Agricultural |
| SH1094 | 15.3 | 3 | J | TX | Agricultural |
| SH1094 | 20.1 | 4 | J | TX | Agricultural |
| SH1094 | 22.2 | 5 | J | TX | Agricultural |
| SH1094 | 32.6 | 6 | J | TX | Agricultural |
| SH1094 | 36.6 | 7 | J | TX | Agricultural |
| SH1094 | 66.8 | 8 | J | TX | Agricultural |
| SH1094 | 13 | 1 | J | TX | Agricultural |
| SH1094 | 15.5 | 2 | J | TX | Agricultural |
| SH1094 | 21.3 | 3 | J | TX | Agricultural |
| SH1094 | 24.6 | 4 | J | TX | Agricultural |
| SH1094 | 28.5 | 5 | J | TX | Agricultural |
| SH1094 | 40.2 | 6 | J | TX | Agricultural |
| SH1094 | 39 | 7 | J | TX | Agricultural |
| SH1094 | 45.7 | 8 | J | TX | Agricultural |
| SH1094 | 10 | 1 | J | TX | Agricultural |
| SH1094 | 13.5 | 2 | J | TX | Agricultural |
| SH1094 | 13.5 | 3 | J | TX | Agricultural |
| SH1094 | 17.9 | 4 | J | TX | Agricultural |
| SH1094 | 27.3 | 5 | J | TX | Agricultural |
| SH1094 | 37.3 | 6 | J | TX | Agricultural |
| SH1094 | 34.3 | 7 | J | TX | Agricultural |
| SH1094 | 55.4 | 8 | J | TX | Agricultural |
| SH1104 | 8 | 1 | J | TX | Agricultural |
| SH1104 | 12.4 | 2 | J | TX | Agricultural |
| SH1104 | 13.3 | 3 | J | TX | Agricultural |
| SH1104 | 17.4 | 4 | J | TX | Agricultural |
| SH1104 | 26.7 | 5 | J | TX | Agricultural |
| SH1104 | 29.5 | 6 | J | TX | Agricultural |
| SH1104 | 42.3 | 7 | J | TX | Agricultural |
| SH1104 | 55.5 | 8 | J | TX | Agricultural |
| SH1104 | 14.6 | 1 | J | TX | Agricultural |
| SH1104 | 15 | 2 | J | TX | Agricultural |
| SH1104 | 15.2 | 3 | J | TX | Agricultural |
| SH1104 | 19 | 4 | J | TX | Agricultural |
| SH1104 | 37.5 | 5 | J | TX | Agricultural |
| SH1104 | 43.8 | 6 | J | TX | Agricultural |
| SH1104 | 62 | 7 | J | TX | Agricultural |
| SH1104 | 51.5 | 8 | J | TX | Agricultural |
| SH1104 | 10 | 1 | J | TX | Agricultural |
| SH1104 | 13.9 | 2 | J | TX | Agricultural |
| SH1104 | 16.7 | 3 | J | TX | Agricultural |
| SH1104 | 19.8 | 4 | J | TX | Agricultural |
| SH1104 | 25.8 | 5 | J | TX | Agricultural |
| SH1104 | 33 | 6 | J | TX | Agricultural |
| SH1104 | 61.1 | 7 | J | TX | Agricultural |
| SH1104 | 8.5 | 1 | J | TX | Agricultural |
| SH1104 | 14 | 2 | J | TX | Agricultural |
| SH1104 | 16.7 | 3 | J | TX | Agricultural |
| SH1104 | 24.7 | 4 | J | TX | Agricultural |
| SH1104 | 32.7 | 5 | J | TX | Agricultural |
| SH1104 | 34.3 | 6 | J | TX | Agricultural |
| SH1104 | 32.7 | 7 | J | TX | Agricultural |
| SH1104 | 47.7 | 8 | J | TX | Agricultural |
| SH1104 | 12.6 | 1 | J | TX | Agricultural |
| SH1104 | 13.8 | 2 | J | TX | Agricultural |
| SH1104 | 14.2 | 3 | J | TX | Agricultural |
| SH1104 | 15.1 | 4 | J | TX | Agricultural |
| SH1104 | 37.2 | 5 | J | TX | Agricultural |
| SH1104 | 57.1 | 6 | J | TX | Agricultural |
| SH1104 | 65.2 | 7 | J | TX | Agricultural |
| SH1116 | 17.8 | 1 | J | TX | Agricultural |
| SH1116 | 14 | 2 | J | TX | Agricultural |
| SH1116 | 27.3 | 3 | J | TX | Agricultural |
| SH1116 | 28.3 | 4 | J | TX | Agricultural |
| SH1116 | 30.2 | 5 | J | TX | Agricultural |
| SH1116 | 35.6 | 6 | J | TX | Agricultural |
| SH1116 | 53.3 | 7 | J | TX | Agricultural |
| SH1116 | 14.8 | 1 | J | TX | Agricultural |
| SH1116 | 32.1 | 2 | J | TX | Agricultural |
| SH1116 | 26.8 | 3 | J | TX | Agricultural |
| SH1116 | 24.9 | 4 | J | TX | Agricultural |
| SH1116 | 18.5 | 5 | J | TX | Agricultural |
| SH1116 | 45.8 | 6 | J | TX | Agricultural |
| SH1116 | 45.8 | 7 | J | TX | Agricultural |
| SH1116 | 15.4 | 1 | J | TX | Agricultural |
| SH1116 | 18.9 | 2 | J | TX | Agricultural |
| SH1116 | 17.3 | 3 | J | TX | Agricultural |
| SH1116 | 20.2 | 4 | J | TX | Agricultural |
| SH1116 | 27.6 | 5 | J | TX | Agricultural |
| SH1116 | 31.8 | 6 | J | TX | Agricultural |
| SH1116 | 45.5 | 7 | J | TX | Agricultural |
| SH1116 | 38.5 | 8 | J | TX | Agricultural |
| SH1116 | 28.4 | 1 | J | TX | Agricultural |
| SH1116 | 42.3 | 2 | J | TX | Agricultural |
| SH1116 | 28.6 | 3 | J | TX | Agricultural |
| SH1116 | 42 | 4 | J | TX | Agricultural |
| SH1116 | 48.3 | 5 | J | TX | Agricultural |
| SH1116 | 23.9 | 6 | J | TX | Agricultural |
| SH1116 | 53.8 | 7 | J | TX | Agricultural |
| SH1116 | 9.9 | 1 | J | TX | Agricultural |
| SH1116 | 16.8 | 2 | J | TX | Agricultural |
| SH1116 | 32.8 | 3 | J | TX | Agricultural |
| SH1116 | 49.3 | 4 | J | TX | Agricultural |
| SH1116 | 59.2 | 5 | J | TX | Agricultural |
| SH1116 | 69.8 | 6 | J | TX | Agricultural |
| SH1116 | 45.9 | 7 | J | TX | Agricultural |
| SH1116 | 8 | 1 | J | TX | Agricultural |
| SH1116 | 18.2 | 2 | J | TX | Agricultural |
| SH1116 | 40.5 | 3 | J | TX | Agricultural |
| SH1116 | 52.4 | 4 | J | TX | Agricultural |
| SH1116 | 62.8 | 5 | J | TX | Agricultural |
| SH1116 | 61.4 | 6 | J | TX | Agricultural |
| SH1116 | 43.3 | 7 | J | TX | Agricultural |
| SH1116 | 39.9 | 8 | J | TX | Agricultural |
| SH1116 | 11 | 1 | J | TX | Agricultural |
| SH1116 | 14.4 | 2 | J | TX | Agricultural |
| SH1116 | 14.6 | 3 | J | TX | Agricultural |
| SH1116 | 71.1 | 4 | J | TX | Agricultural |
| SH1116 | 27.6 | 5 | J | TX | Agricultural |
| SH1116 | 40.1 | 6 | J | TX | Agricultural |
| SH1116 | 58.8 | 7 | J | TX | Agricultural |
| SH1126 | 14.6 | 1 | J | TX |  |
| SH1126 | 16.8 | 2 | J | TX |  |
| SH1126 | 18.9 | 3 | J | TX |  |
| SH1126 | 25.1 | 4 | J | TX |  |
| SH1126 | 25.1 | 5 | J | TX |  |
| SH1126 | 26.7 | 6 | J | TX |  |
| SH1126 | 28.8 | 7 | J | TX |  |
| SH1126 | 59.1 | 8 | J | TX |  |
| SH1126 | 15.8 | 1 | J | TX |  |
| SH1126 | 19.5 | 2 | J | TX |  |
| SH1126 | 23 | 3 | J | TX |  |
| SH1126 | 31.6 | 4 | J | TX |  |
| SH1126 | 31.6 | 5 | J | TX |  |
| SH1126 | 33.6 | 6 | J | TX |  |
| SH1126 | 35.7 | 7 | J | TX |  |
| SH1126 | 63.1 | 8 | J | TX |  |
| SH1126 | 13.4 | 1 | J | TX |  |
| SH1126 | 17.1 | 2 | J | TX |  |
| SH1126 | 38.9 | 3 | J | TX |  |
| SH1126 | 25.8 | 4 | J | TX |  |
| SH1126 | 31.7 | 5 | J | TX |  |
| SH1126 | 40 | 6 | J | TX |  |
| SH1126 | 71.5 | 7 | J | TX |  |
| SH1126 | 7 | 1 | J | TX |  |
| SH1126 | 14.5 | 2 | J | TX |  |
| SH1126 | 15.4 | 3 | J | TX |  |
| SH1126 | 15.7 | 4 | J | TX |  |
| SH1126 | 17.9 | 5 | J | TX |  |
| SH1126 | 21.1 | 6 | J | TX |  |
| SH1126 | 28.9 | 7 | J | TX |  |
| SH1126 | 35.2 | 8 | J | TX |  |
| SH1136 | 12.9 | 1 | J | NM | Roadside |
| SH1136 | 31.7 | 2 | J | NM | Roadside |
| SH1136 | 29.7 | 3 | J | NM | Roadside |
| SH1136 | 29.7 | 4 | J | NM | Roadside |
| SH1136 | 52.7 | 5 | J | NM | Roadside |
| SH1136 | 7 | 1 | J | NM | Roadside |
| SH1136 | 39 | 2 | J | NM | Roadside |
| SH1136 | 39 | 3 | J | NM | Roadside |
| SH1136 | 32.9 | 4 | J | NM | Roadside |
| SH1136 | 32.9 | 5 | J | NM | Roadside |
| SH1136 | 77.6 | 6 | J | NM | Roadside |
| SH1136 | 74.2 | 7 | J | NM | Roadside |
| SH1136 | 75.2 | 8 | J | NM | Roadside |
| SH1136 | 13.9 | 1 | J | NM | Roadside |
| SH1136 | 14.2 | 2 | J | NM | Roadside |
| SH1136 | 19 | 3 | J | NM | Roadside |
| SH1136 | 15.7 | 4 | J | NM | Roadside |
| SH1136 | 21.5 | 5 | J | NM | Roadside |
| SH1136 | 30.2 | 6 | J | NM | Roadside |
| SH1136 | 44.8 | 7 | J | NM | Roadside |
| SH1136 | 20.8 | 8 | J | NM | Roadside |
| SH1136 | 18.3 | 1 | J | NM | Roadside |
| SH1136 | 22 | 2 | J | NM | Roadside |
| SH1136 | 16.1 | 3 | J | NM | Roadside |
| SH1136 | 18.1 | 4 | J | NM | Roadside |
| SH1136 | 16.1 | 5 | J | NM | Roadside |
| SH1136 | 25 | 6 | J | NM | Roadside |
| SH1136 | 27.6 | 7 | J | NM | Roadside |
| SH1136 | 49.7 | 8 | J | NM | Roadside |
| SH1136 | 13.1 | 1 | J | NM | Roadside |
| SH1136 | 18.1 | 2 | J | NM | Roadside |
| SH1136 | 21.1 | 3 | J | NM | Roadside |
| SH1136 | 15.5 | 4 | J | NM | Roadside |
| SH1136 | 20.7 | 5 | J | NM | Roadside |
| SH1136 | 20.3 | 6 | J | NM | Roadside |
| SH1136 | 68.1 | 7 | J | NM | Roadside |
| SH1136 | 67.5 | 8 | J | NM | Roadside |
| SH1136 | 8 | 1 | J | NM | Roadside |
| SH1136 | 16 | 2 | J | NM | Roadside |
| SH1136 | 24.8 | 3 | J | NM | Roadside |
| SH1136 | 21.4 | 4 | J | NM | Roadside |
| SH1136 | 21.4 | 5 | J | NM | Roadside |
| SH1136 | 18.4 | 6 | J | NM | Roadside |
| SH1136 | 31.4 | 7 | J | NM | Roadside |
| SH1136 | 68.5 | 8 | J | NM | Roadside |
| SH1136 | 14.2 | 1 | J | NM | Roadside |
| SH1136 | 15.4 | 2 | J | NM | Roadside |
| SH1136 | 52.4 | 3 | J | NM | Roadside |
| SH1136 | 27.2 | 4 | J | NM | Roadside |
| SH1136 | 27.2 | 5 | J | NM | Roadside |
| SH1136 | 37.1 | 6 | J | NM | Roadside |
| SH1136 | 63.1 | 7 | J | NM | Roadside |
| SH1136 | 53.7 | 1 | J | NM | Roadside |
| SH1136 | 28.6 | 2 | J | NM | Roadside |
| SH1136 | 43.3 | 3 | J | NM | Roadside |
| SH1136 | 23.4 | 4 | J | NM | Roadside |
| SH1136 | 27.2 | 5 | J | NM | Roadside |
| SH1136 | 33.3 | 6 | J | NM | Roadside |
| SH1136 | 48.3 | 7 | J | NM | Roadside |
| SH1136 | 56.4 | 8 | J | NM | Roadside |
| SH1136 | 69.2 | 1 | J | NM | Roadside |
| SH1136 | 46.7 | 2 | J | NM | Roadside |
| SH1136 | 47.5 | 3 | J | NM | Roadside |
| SH1136 | 46.1 | 4 | J | NM | Roadside |
| SH1136 | 68.5 | 5 | J | NM | Roadside |
| SH1136 | 66 | 6 | J | NM | Roadside |
| SH1136 | 16.7 | 1 | J | NM | Roadside |
| SH1136 | 22.4 | 2 | J | NM | Roadside |
| SH1136 | 30.6 | 3 | J | NM | Roadside |
| SH1136 | 50.7 | 4 | J | NM | Roadside |
| SH1136 | 31 | 5 | J | NM | Roadside |
| SH1136 | 28.1 | 6 | J | NM | Roadside |
| SH1136 | 43.2 | 7 | J | NM | Roadside |
| SH1136 | 30.9 | 8 | J | NM | Roadside |
| SH1136 | 17.3 | 1 | J | NM | Roadside |
| SH1136 | 23.3 | 2 | J | NM | Roadside |
| SH1136 | 27.5 | 3 | J | NM | Roadside |
| SH1136 | 38.8 | 4 | J | NM | Roadside |
| SH1136 | 27.8 | 5 | J | NM | Roadside |
| SH1136 | 28.2 | 6 | J | NM | Roadside |
| SH1136 | 30.3 | 7 | J | NM | Roadside |
| SH1136 | 49.7 | 1 | J | NM | Roadside |
| SH1136 | 46.8 | 2 | J | NM | Roadside |
| SH1136 | 38.6 | 3 | J | NM | Roadside |
| SH1136 | 40.3 | 4 | J | NM | Roadside |
| SH1136 | 52.1 | 5 | J | NM | Roadside |
| SH1136 | 65.5 | 6 | J | NM | Roadside |
| SH1136 | 72.1 | 7 | J | NM | Roadside |
| SH1136 | 9.5 | 1 | J | NM | Roadside |
| SH1136 | 11.7 | 2 | J | NM | Roadside |
| SH1136 | 15.6 | 3 | J | NM | Roadside |
| SH1136 | 20.1 | 4 | J | NM | Roadside |
| SH1136 | 33.9 | 5 | J | NM | Roadside |
| SH1136 | 44.4 | 6 | J | NM | Roadside |
| SH1136 | 77.9 | 7 | J | NM | Roadside |
| SH1152 | 17.3 | 1 | J | GA | Undisturbed |
| SH1152 | 19 | 2 | J | GA | Undisturbed |
| SH1152 | 20.8 | 3 | J | GA | Undisturbed |
| SH1152 | 36.2 | 4 | J | GA | Undisturbed |
| SH1152 | 40.9 | 5 | J | GA | Undisturbed |
| SH1152 | 40.9 | 6 | J | GA | Undisturbed |
| SH1152 | 69.2 | 7 | J | GA | Undisturbed |
| SH1152 | 69.2 | 8 | J | GA | Undisturbed |
| SH1152 | 36.5 | 1 | J | GA | Undisturbed |
| SH1152 | 44.3 | 2 | J | GA | Undisturbed |
| SH1152 | 25.4 | 3 | J | GA | Undisturbed |
| SH1152 | 13.6 | 4 | J | GA | Undisturbed |
| SH1152 | 36.4 | 5 | J | GA | Undisturbed |
| SH1152 | 8.5 | 6 | J | GA | Undisturbed |
| SH1152 | 19.3 | 7 | J | GA | Undisturbed |
| SH1152 | 46.8 | 8 | J | GA | Undisturbed |
| SH1152 | 14.8 | 1 | J | GA | Undisturbed |
| SH1152 | 18.1 | 2 | J | GA | Undisturbed |
| SH1152 | 20.4 | 3 | J | GA | Undisturbed |
| SH1152 | 25.5 | 4 | J | GA | Undisturbed |
| SH1152 | 32.7 | 5 | J | GA | Undisturbed |
| SH1152 | 28.8 | 6 | J | GA | Undisturbed |
| SH1152 | 50.6 | 7 | J | GA | Undisturbed |
| SH1152 | 28.5 | 8 | J | GA | Undisturbed |
| SH1152 | 23.8 | 1 | J | GA | Undisturbed |
| SH1152 | 32.8 | 2 | J | GA | Undisturbed |
| SH1152 | 26.7 | 3 | J | GA | Undisturbed |
| SH1152 | 65.6 | 4 | J | GA | Undisturbed |
| SH1152 | 34 | 5 | J | GA | Undisturbed |
| SH1152 | 34.5 | 6 | J | GA | Undisturbed |
| SH1152 | 68.6 | 1 | J | GA | Undisturbed |
| SH1152 | 58 | 2 | J | GA | Undisturbed |
| SH1152 | 63.7 | 3 | J | GA | Undisturbed |
| SH1152 | 27.7 | 4 | J | GA | Undisturbed |
| SH1152 | 31.5 | 5 | J | GA | Undisturbed |
| SH1152 | 41.1 | 6 | J | GA | Undisturbed |
| SH1152 | 56.6 | 7 | J | GA | Undisturbed |
| SH1152 | 8 | 1 | J | GA | Undisturbed |
| SH1152 | 13.5 | 2 | J | GA | Undisturbed |
| SH1152 | 34.1 | 3 | J | GA | Undisturbed |
| SH1152 | 47.5 | 4 | J | GA | Undisturbed |
| SH1152 | 37.2 | 5 | J | GA | Undisturbed |
| SH1152 | 50.9 | 6 | J | GA | Undisturbed |
| SH1152 | 37.8 | 7 | J | GA | Undisturbed |
| SH1152 | 33.7 | 1 | J | GA | Undisturbed |
| SH1152 | 26 | 2 | J | GA | Undisturbed |
| SH1152 | 30.3 | 3 | J | GA | Undisturbed |
| SH1152 | 32.5 | 4 | J | GA | Undisturbed |
| SH1152 | 41.7 | 5 | J | GA | Undisturbed |
| SH1152 | 45.6 | 6 | J | GA | Undisturbed |
| SH1154 | 12.6 | 1 | J | GA | Undisturbed |
| SH1154 | 12.6 | 2 | J | GA | Undisturbed |
| SH1154 | 14.2 | 3 | J | GA | Undisturbed |
| SH1154 | 37.4 | 4 | J | GA | Undisturbed |
| SH1154 | 75.1 | 5 | J | GA | Undisturbed |
| SH1154 | 80.2 | 6 | J | GA | Undisturbed |
| SH1154 | 80.2 | 7 | J | GA | Undisturbed |
| SH1154 | 10 | 1 | J | GA | Undisturbed |
| SH1154 | 15.2 | 2 | J | GA | Undisturbed |
| SH1154 | 18.6 | 3 | J | GA | Undisturbed |
| SH1154 | 27.3 | 4 | J | GA | Undisturbed |
| SH1154 | 34.4 | 5 | J | GA | Undisturbed |
| SH1154 | 18.7 | 6 | J | GA | Undisturbed |
| SH1154 | 25.5 | 7 | J | GA | Undisturbed |
| SH1154 | 63.9 | 8 | J | GA | Undisturbed |
| SH1154 | 11 | 1 | J | GA | Undisturbed |
| SH1154 | 16.2 | 2 | J | GA | Undisturbed |
| SH1154 | 14.7 | 3 | J | GA | Undisturbed |
| SH1154 | 29.7 | 4 | J | GA | Undisturbed |
| SH1154 | 46.6 | 5 | J | GA | Undisturbed |
| SH1154 | 73.2 | 6 | J | GA | Undisturbed |
| SH1154 | 51.3 | 7 | J | GA | Undisturbed |
| SH1154 | 64.6 | 8 | J | GA | Undisturbed |
| SH1154 | 7 | 1 | J | GA | Undisturbed |
| SH1154 | 14 | 2 | J | GA | Undisturbed |
| SH1154 | 15.6 | 3 | J | GA | Undisturbed |
| SH1154 | 29.6 | 4 | J | GA | Undisturbed |
| SH1154 | 35.6 | 5 | J | GA | Undisturbed |
| SH1154 | 40.1 | 6 | J | GA | Undisturbed |
| SH1154 | 12 | 1 | J | GA | Undisturbed |
| SH1154 | 12.6 | 2 | J | GA | Undisturbed |
| SH1154 | 12.7 | 3 | J | GA | Undisturbed |
| SH1154 | 27.4 | 4 | J | GA | Undisturbed |
| SH1154 | 25.3 | 5 | J | GA | Undisturbed |
| SH1154 | 24.1 | 6 | J | GA | Undisturbed |
| SH1154 | 12.5 | 1 | J | GA | Undisturbed |
| SH1154 | 13.7 | 2 | J | GA | Undisturbed |
| SH1154 | 20.3 | 3 | J | GA | Undisturbed |
| SH1154 | 29.4 | 4 | J | GA | Undisturbed |
| SH1154 | 36.1 | 5 | J | GA | Undisturbed |
| SH1154 | 52.7 | 6 | J | GA | Undisturbed |
| SH1154 | 15.3 | 1 | J | GA | Undisturbed |
| SH1154 | 17.6 | 2 | J | GA | Undisturbed |
| SH1154 | 22.5 | 3 | J | GA | Undisturbed |
| SH1154 | 22.5 | 4 | J | GA | Undisturbed |
| SH1154 | 32.7 | 5 | J | GA | Undisturbed |
| SH1154 | 68.6 | 6 | J | GA | Undisturbed |
| SH1154 | 0 | 1 | J | GA | Undisturbed |
| SH1154 | 14.7 | 2 | J | GA | Undisturbed |
| SH1154 | 13.5 | 3 | J | GA | Undisturbed |
| SH1154 | 15.6 | 4 | J | GA | Undisturbed |
| SH1154 | 16.8 | 5 | J | GA | Undisturbed |
| SH1154 | 24.7 | 6 | J | GA | Undisturbed |
| SH1154 | 46.8 | 7 | J | GA | Undisturbed |
| SH1154 | 73.7 | 8 | J | GA | Undisturbed |
| SH1154 | 13.2 | 1 | J | GA | Undisturbed |
| SH1154 | 16.3 | 2 | J | GA | Undisturbed |
| SH1154 | 19.4 | 3 | J | GA | Undisturbed |
| SH1154 | 26 | 4 | J | GA | Undisturbed |
| SH1154 | 40.1 | 5 | J | GA | Undisturbed |
| SH1154 | 32.2 | 6 | J | GA | Undisturbed |
| SH1154 | 48.4 | 7 | J | GA | Undisturbed |
| SH1154 | 6 | 1 | J | GA | Undisturbed |
| SH1154 | 13.7 | 2 | J | GA | Undisturbed |
| SH1154 | 19.1 | 3 | J | GA | Undisturbed |
| SH1154 | 20.4 | 4 | J | GA | Undisturbed |
| SH1154 | 17.4 | 5 | J | GA | Undisturbed |
| SH1154 | 19.8 | 6 | J | GA | Undisturbed |
| SH1154 | 21.8 | 7 | J | GA | Undisturbed |
| SH1154 | 40.7 | 8 | J | GA | Undisturbed |
| SH1154 | 8 | 1 | J | GA | Undisturbed |
| SH1154 | 15.7 | 2 | J | GA | Undisturbed |
| SH1154 | 19.3 | 3 | J | GA | Undisturbed |
| SH1154 | 19.4 | 4 | J | GA | Undisturbed |
| SH1154 | 20.2 | 5 | J | GA | Undisturbed |
| SH1154 | 21.9 | 6 | J | GA | Undisturbed |
| SH1154 | 33.6 | 7 | J | GA | Undisturbed |
| SH1154 | 30.6 | 8 | J | GA | Undisturbed |
| SH1154 | 14.1 | 1 | J | GA | Undisturbed |
| SH1154 | 17.4 | 2 | J | GA | Undisturbed |
| SH1154 | 31.7 | 3 | J | GA | Undisturbed |
| SH1154 | 24 | 4 | J | GA | Undisturbed |
| SH1154 | 16.8 | 5 | J | GA | Undisturbed |
| SH1154 | 30.8 | 6 | J | GA | Undisturbed |
| SH1154 | 29.1 | 7 | J | GA | Undisturbed |
| SH1154 | 13.2 | 1 | J | GA | Undisturbed |
| SH1154 | 13.6 | 2 | J | GA | Undisturbed |
| SH1154 | 17.8 | 3 | J | GA | Undisturbed |
| SH1154 | 16.8 | 4 | J | GA | Undisturbed |
| SH1154 | 38.2 | 5 | J | GA | Undisturbed |
| SH1154 | 65.9 | 6 | J | GA | Undisturbed |
| SH1154 | 65.9 | 7 | J | GA | Undisturbed |
| SH1154 | 12.1 | 1 | J | GA | Undisturbed |
| SH1154 | 14.1 | 2 | J | GA | Undisturbed |
| SH1154 | 14.4 | 3 | J | GA | Undisturbed |
| SH1154 | 13.7 | 4 | J | GA | Undisturbed |
| SH1154 | 18.3 | 5 | J | GA | Undisturbed |
| SH1154 | 58.4 | 6 | J | GA | Undisturbed |
| SH1154 | 57.8 | 7 | J | GA | Undisturbed |
| SH1165 | 13.1 | 1 | J | GA | Roadside |
| SH1165 | 12.8 | 2 | J | GA | Roadside |
| SH1165 | 26 | 3 | J | GA | Roadside |
| SH1165 | 29.7 | 4 | J | GA | Roadside |
| SH1165 | 34.5 | 5 | J | GA | Roadside |
| SH1165 | 91.1 | 6 | J | GA | Roadside |
| SH1165 | 20.9 | 1 | J | GA | Roadside |
| SH1165 | 22.7 | 2 | J | GA | Roadside |
| SH1165 | 24.1 | 3 | J | GA | Roadside |
| SH1165 | 29 | 4 | J | GA | Roadside |
| SH1165 | 30.6 | 5 | J | GA | Roadside |
| SH1165 | 38.4 | 6 | J | GA | Roadside |
| SH1165 | 81.4 | 7 | J | GA | Roadside |
| SH1165 | 81.4 | 8 | J | GA | Roadside |
| SH1165 | 17.9 | 1 | J | GA | Roadside |
| SH1165 | 40.8 | 2 | J | GA | Roadside |
| SH1165 | 19.2 | 3 | J | GA | Roadside |
| SH1165 | 16.2 | 4 | J | GA | Roadside |
| SH1165 | 17.8 | 5 | J | GA | Roadside |
| SH1165 | 29.9 | 6 | J | GA | Roadside |
| SH1165 | 40.2 | 7 | J | GA | Roadside |
| SH1165 | 72 | 8 | J | GA | Roadside |
| SH1165 | 13.9 | 1 | J | GA | Roadside |
| SH1165 | 15.2 | 2 | J | GA | Roadside |
| SH1165 | 22.4 | 3 | J | GA | Roadside |
| SH1165 | 19.4 | 4 | J | GA | Roadside |
| SH1165 | 42.7 | 5 | J | GA | Roadside |
| SH1165 | 42.7 | 6 | J | GA | Roadside |
| SH1165 | 46.5 | 7 | J | GA | Roadside |
| SH1165 | 46.6 | 1 | J | GA | Roadside |
| SH1165 | 38.5 | 2 | J | GA | Roadside |
| SH1165 | 26.3 | 3 | J | GA | Roadside |
| SH1165 | 26.4 | 4 | J | GA | Roadside |
| SH1165 | 36.3 | 5 | J | GA | Roadside |
| SH1165 | 38 | 6 | J | GA | Roadside |
| SH1165 | 15.7 | 1 | J | GA | Roadside |
| SH1165 | 15.2 | 2 | J | GA | Roadside |
| SH1165 | 21.9 | 3 | J | GA | Roadside |
| SH1165 | 41.3 | 4 | J | GA | Roadside |
| SH1165 | 54.7 | 5 | J | GA | Roadside |
| SH1165 | 50 | 6 | J | GA | Roadside |
| SH1165 | 58.4 | 7 | J | GA | Roadside |
| SH1165 | 58.3 | 1 | J | GA | Roadside |
| SH1165 | 46.3 | 2 | J | GA | Roadside |
| SH1165 | 26.4 | 3 | J | GA | Roadside |
| SH1165 | 22.8 | 4 | J | GA | Roadside |
| SH1165 | 32.8 | 5 | J | GA | Roadside |
| SH1165 | 36.6 | 1 | J | GA | Roadside |
| SH1165 | 55.3 | 2 | J | GA | Roadside |
| SH1165 | 30 | 3 | J | GA | Roadside |
| SH1165 | 46.2 | 4 | J | GA | Roadside |
| SH1165 | 42.1 | 5 | J | GA | Roadside |
| SH1165 | 68.2 | 6 | J | GA | Roadside |
| SH1165 | 55.6 | 7 | J | GA | Roadside |
| SH1165 | 22.4 | 8 | J | GA | Roadside |
| SH1165 | 12.2 | 1 | J | GA | Roadside |
| SH1165 | 13.6 | 2 | J | GA | Roadside |
| SH1165 | 16.8 | 3 | J | GA | Roadside |
| SH1165 | 24.7 | 4 | J | GA | Roadside |
| SH1165 | 31.3 | 5 | J | GA | Roadside |
| SH1165 | 41.1 | 6 | J | GA | Roadside |
| SH1165 | 10 | 1 | J | GA | Roadside |
| SH1165 | 16.3 | 2 | J | GA | Roadside |
| SH1165 | 48.9 | 3 | J | GA | Roadside |
| SH1165 | 36.5 | 4 | J | GA | Roadside |
| SH1165 | 21.4 | 5 | J | GA | Roadside |
| SH1165 | 23.4 | 6 | J | GA | Roadside |
| SH1165 | 54 | 7 | J | GA | Roadside |
| SH1165 | 8 | 1 | J | GA | Roadside |
| SH1165 | 15.9 | 2 | J | GA | Roadside |
| SH1165 | 32.6 | 3 | J | GA | Roadside |
| SH1165 | 20.6 | 4 | J | GA | Roadside |
| SH1165 | 29.2 | 5 | J | GA | Roadside |
| SH1165 | 42.7 | 6 | J | GA | Roadside |
| SH1165 | 45.9 | 7 | J | GA | Roadside |
| SH1201 | 20.3 | 1 | J | TX | Undisturbed |
| SH1201 | 26.5 | 2 | J | TX | Undisturbed |
| SH1201 | 17.4 | 3 | J | TX | Undisturbed |
| SH1201 | 15.6 | 4 | J | TX | Undisturbed |
| SH1201 | 15.4 | 5 | J | TX | Undisturbed |
| SH1201 | 18.9 | 6 | J | TX | Undisturbed |
| SH1201 | 29.9 | 7 | J | TX | Undisturbed |
| SH1201 | 47.7 | 8 | J | TX | Undisturbed |
| SH1201 | 12.6 | 1 | J | TX | Undisturbed |
| SH1201 | 16.1 | 2 | J | TX | Undisturbed |
| SH1201 | 25.1 | 3 | J | TX | Undisturbed |
| SH1201 | 15.5 | 4 | J | TX | Undisturbed |
| SH1201 | 16.1 | 5 | J | TX | Undisturbed |
| SH1201 | 17.4 | 6 | J | TX | Undisturbed |
| SH1201 | 19.6 | 7 | J | TX | Undisturbed |
| SH1201 | 23.8 | 8 | J | TX | Undisturbed |
| SH1201 | 11.6 | 1 | J | TX | Undisturbed |
| SH1201 | 14.4 | 2 | J | TX | Undisturbed |
| SH1201 | 21.6 | 3 | J | TX | Undisturbed |
| SH1201 | 20.7 | 4 | J | TX | Undisturbed |
| SH1201 | 20.7 | 5 | J | TX | Undisturbed |
| SH1201 | 37.2 | 6 | J | TX | Undisturbed |
| SH1201 | 22.5 | 7 | J | TX | Undisturbed |
| SH1201 | 26 | 8 | J | TX | Undisturbed |
| SH1201 | 5 | 1 | J | TX | Undisturbed |
| SH1201 | 18.4 | 2 | J | TX | Undisturbed |
| SH1201 | 22.8 | 3 | J | TX | Undisturbed |
| SH1201 | 24.1 | 4 | J | TX | Undisturbed |
| SH1201 | 16.5 | 5 | J | TX | Undisturbed |
| SH1201 | 25.6 | 6 | J | TX | Undisturbed |
| SH1201 | 16.7 | 7 | J | TX | Undisturbed |
| SH1201 | 23 | 8 | J | TX | Undisturbed |
| SH1201 | 17.1 | 1 | J | TX | Undisturbed |
| SH1201 | 19.1 | 2 | J | TX | Undisturbed |
| SH1201 | 23.4 | 3 | J | TX | Undisturbed |
| SH1201 | 23.4 | 4 | J | TX | Undisturbed |
| SH1201 | 33.4 | 5 | J | TX | Undisturbed |
| SH1201 | 35.2 | 6 | J | TX | Undisturbed |
| SH1201 | 36.9 | 7 | J | TX | Undisturbed |
| SH1201 | 36.9 | 8 | J | TX | Undisturbed |
| SH1201 | 21 | 1 | J | TX | Undisturbed |
| SH1201 | 34.9 | 2 | J | TX | Undisturbed |
| SH1201 | 19.3 | 3 | J | TX | Undisturbed |
| SH1201 | 31.2 | 4 | J | TX | Undisturbed |
| SH1201 | 19.6 | 5 | J | TX | Undisturbed |
| SH1201 | 29.1 | 6 | J | TX | Undisturbed |
| SH1201 | 18.1 | 7 | J | TX | Undisturbed |
| SH1201 | 21.4 | 8 | J | TX | Undisturbed |
| SH1201 | 7.9 | 1 | J | TX | Undisturbed |
| SH1201 | 15.5 | 2 | J | TX | Undisturbed |
| SH1201 | 19.7 | 3 | J | TX | Undisturbed |
| SH1201 | 15.1 | 4 | J | TX | Undisturbed |
| SH1201 | 25 | 5 | J | TX | Undisturbed |
| SH1201 | 19.7 | 6 | J | TX | Undisturbed |
| SH1201 | 22.3 | 7 | J | TX | Undisturbed |
| SH1201 | 23.3 | 8 | J | TX | Undisturbed |
| SH1201 | 12.4 | 1 | J | TX | Undisturbed |
| SH1201 | 18.4 | 2 | J | TX | Undisturbed |
| SH1201 | 27.9 | 3 | J | TX | Undisturbed |
| SH1201 | 28.5 | 4 | J | TX | Undisturbed |
| SH1201 | 19.5 | 5 | J | TX | Undisturbed |
| SH1201 | 25.5 | 6 | J | TX | Undisturbed |
| SH1201 | 30.4 | 7 | J | TX | Undisturbed |
| SH1201 | 24.4 | 8 | J | TX | Undisturbed |
| SH1201 | 10.6 | 1 | J | TX | Undisturbed |
| SH1201 | 12.7 | 2 | J | TX | Undisturbed |
| SH1201 | 14.9 | 3 | J | TX | Undisturbed |
| SH1201 | 25.3 | 4 | J | TX | Undisturbed |
| SH1201 | 33.9 | 5 | J | TX | Undisturbed |
| SH1201 | 50.6 | 6 | J | TX | Undisturbed |
| SH1201 | 50.6 | 7 | J | TX | Undisturbed |
| SH1201 | 79.5 | 8 | J | TX | Undisturbed |
| SH1229 | 8 | 1 | J | TX | Roadside |
| SH1229 | 13.7 | 2 | J | TX | Roadside |
| SH1229 | 16.8 | 3 | J | TX | Roadside |
| SH1229 | 31.8 | 4 | J | TX | Roadside |
| SH1229 | 43.1 | 5 | J | TX | Roadside |
| SH1229 | 43.1 | 6 | J | TX | Roadside |
| SH1229 | 57.1 | 7 | J | TX | Roadside |
| SH1229 | 31.7 | 8 | J | TX | Roadside |
| SH1229 | 10 | 1 | J | TX | Roadside |
| SH1229 | 17.8 | 2 | J | TX | Roadside |
| SH1229 | 18.9 | 3 | J | TX | Roadside |
| SH1229 | 13.4 | 4 | J | TX | Roadside |
| SH1229 | 19.9 | 5 | J | TX | Roadside |
| SH1229 | 33.1 | 6 | J | TX | Roadside |
| SH1229 | 26 | 7 | J | TX | Roadside |
| SH1229 | 40.8 | 8 | J | TX | Roadside |
| SH1229 | 14 | 1 | J | TX | Roadside |
| SH1229 | 17.6 | 2 | J | TX | Roadside |
| SH1229 | 32 | 3 | J | TX | Roadside |
| SH1229 | 26.6 | 4 | J | TX | Roadside |
| SH1229 | 24.8 | 5 | J | TX | Roadside |
| SH1229 | 30 | 6 | J | TX | Roadside |
| SH1229 | 38.7 | 7 | J | TX | Roadside |
| SH1229 | 61.3 | 8 | J | TX | Roadside |
| SH1229 | 18.2 | 1 | J | TX | Roadside |
| SH1229 | 26.8 | 2 | J | TX | Roadside |
| SH1229 | 26.4 | 3 | J | TX | Roadside |
| SH1229 | 27.8 | 4 | J | TX | Roadside |
| SH1229 | 31.1 | 5 | J | TX | Roadside |
| SH1229 | 25.9 | 6 | J | TX | Roadside |
| SH1229 | 18.2 | 7 | J | TX | Roadside |
| SH1229 | 15.4 | 1 | J | TX | Roadside |
| SH1229 | 19.1 | 2 | J | TX | Roadside |
| SH1229 | 19.6 | 3 | J | TX | Roadside |
| SH1229 | 21.9 | 4 | J | TX | Roadside |
| SH1229 | 31.1 | 5 | J | TX | Roadside |
| SH1229 | 33.4 | 6 | J | TX | Roadside |
| SH1229 | 53.6 | 7 | J | TX | Roadside |
| SH1229 | 30.4 | 8 | J | TX | Roadside |
| SH1229 | 16.3 | 1 | J | TX | Roadside |
| SH1229 | 22 | 2 | J | TX | Roadside |
| SH1229 | 27.1 | 3 | J | TX | Roadside |
| SH1229 | 22.3 | 4 | J | TX | Roadside |
| SH1229 | 14.2 | 5 | J | TX | Roadside |
| SH1229 | 19.3 | 6 | J | TX | Roadside |
| SH1229 | 40.1 | 7 | J | TX | Roadside |
| SH1229 | 73.3 | 8 | J | TX | Roadside |
| SH1229 | 10 | 1 | J | TX | Roadside |
| SH1229 | 16.7 | 2 | J | TX | Roadside |
| SH1229 | 23.8 | 3 | J | TX | Roadside |
| SH1229 | 17.8 | 4 | J | TX | Roadside |
| SH1229 | 20.1 | 5 | J | TX | Roadside |
| SH1229 | 33.6 | 6 | J | TX | Roadside |
| SH1229 | 52.8 | 7 | J | TX | Roadside |
| SH1229 | 52.8 | 8 | J | TX | Roadside |
| SH1229 | 14.2 | 1 | J | TX | Roadside |
| SH1229 | 20.5 | 2 | J | TX | Roadside |
| SH1229 | 30.1 | 3 | J | TX | Roadside |
| SH1229 | 22.6 | 4 | J | TX | Roadside |
| SH1229 | 30.5 | 5 | J | TX | Roadside |
| SH1229 | 28.4 | 6 | J | TX | Roadside |
| SH1229 | 63.9 | 7 | J | TX | Roadside |
| SH1229 | 10 | 1 | J | TX | Roadside |
| SH1229 | 15.6 | 2 | J | TX | Roadside |
| SH1229 | 27.2 | 3 | J | TX | Roadside |
| SH1229 | 29 | 4 | J | TX | Roadside |
| SH1229 | 23.7 | 5 | J | TX | Roadside |
| SH1229 | 43.2 | 6 | J | TX | Roadside |
| SH1229 | 22.2 | 7 | J | TX | Roadside |
| SH1229 | 26.7 | 8 | J | TX | Roadside |
| SH1233 | 18.2 | 1 | J | TX | Roadside |
| SH1233 | 27.2 | 2 | J | TX | Roadside |
| SH1233 | 32.3 | 3 | J | TX | Roadside |
| SH1233 | 28.2 | 4 | J | TX | Roadside |
| SH1233 | 35.6 | 5 | J | TX | Roadside |
| SH1233 | 49 | 6 | J | TX | Roadside |
| SH1233 | 52.8 | 7 | J | TX | Roadside |
| SH1233 | 80 | 8 | J | TX | Roadside |
| SH1233 | 5 | 1 | J | TX | Roadside |
| SH1233 | 12.4 | 2 | J | TX | Roadside |
| SH1233 | 24 | 3 | J | TX | Roadside |
| SH1233 | 26 | 4 | J | TX | Roadside |
| SH1233 | 71.5 | 5 | J | TX | Roadside |
| SH1233 | 71.5 | 6 | J | TX | Roadside |
| SH1233 | 7 | 1 | J | TX | Roadside |
| SH1233 | 14.7 | 2 | J | TX | Roadside |
| SH1233 | 15 | 3 | J | TX | Roadside |
| SH1233 | 22.9 | 4 | J | TX | Roadside |
| SH1233 | 46.9 | 5 | J | TX | Roadside |
| SH1233 | 48 | 6 | J | TX | Roadside |
| SH1247 | 14.9 | 1 | J | KS | Disturbed |
| SH1247 | 36.4 | 2 | J | KS | Disturbed |
| SH1247 | 29.4 | 3 | J | KS | Disturbed |
| SH1247 | 43.3 | 4 | J | KS | Disturbed |
| SH1247 | 43.3 | 5 | J | KS | Disturbed |
| SH1247 | 26.5 | 6 | J | KS | Disturbed |
| SH1247 | 21.3 | 7 | J | KS | Disturbed |
| SH1247 | 20.1 | 1 | J | KS | Disturbed |
| SH1247 | 48.1 | 2 | J | KS | Disturbed |
| SH1247 | 22.1 | 3 | J | KS | Disturbed |
| SH1247 | 28.6 | 4 | J | KS | Disturbed |
| SH1247 | 35.8 | 5 | J | KS | Disturbed |
| SH1247 | 45.6 | 6 | J | KS | Disturbed |
| SH1247 | 15.3 | 1 | J | KS | Disturbed |
| SH1247 | 26.3 | 2 | J | KS | Disturbed |
| SH1247 | 31 | 3 | J | KS | Disturbed |
| SH1247 | 23.4 | 4 | J | KS | Disturbed |
| SH1247 | 32.5 | 5 | J | KS | Disturbed |
| SH1247 | 34.7 | 6 | J | KS | Disturbed |
| SH1247 | 33.5 | 7 | J | KS | Disturbed |
| SH1247 | 67.3 | 8 | J | KS | Disturbed |
| SH1247 | 7 | 1 | J | KS | Disturbed |
| SH1247 | 13.2 | 2 | J | KS | Disturbed |
| SH1247 | 18.6 | 3 | J | KS | Disturbed |
| SH1247 | 21.2 | 4 | J | KS | Disturbed |
| SH1247 | 41.5 | 5 | J | KS | Disturbed |
| SH1247 | 52.7 | 6 | J | KS | Disturbed |
| SH1247 | 52.7 | 7 | J | KS | Disturbed |
| SH1247 | 83.6 | 8 | J | KS | Disturbed |
| SH1247 | 17.3 | 1 | J | KS | Disturbed |
| SH1247 | 18.8 | 2 | J | KS | Disturbed |
| SH1247 | 18.9 | 3 | J | KS | Disturbed |
| SH1247 | 30.3 | 4 | J | KS | Disturbed |
| SH1247 | 34.3 | 5 | J | KS | Disturbed |
| SH1247 | 53.3 | 6 | J | KS | Disturbed |
| SH1247 | 87.3 | 7 | J | KS | Disturbed |
| SH1247 | 22 | 1 | J | KS | Disturbed |
| SH1247 | 27.4 | 2 | J | KS | Disturbed |
| SH1247 | 19.2 | 3 | J | KS | Disturbed |
| SH1247 | 19 | 4 | J | KS | Disturbed |
| SH1247 | 30.1 | 5 | J | KS | Disturbed |
| SH1247 | 39.1 | 6 | J | KS | Disturbed |
| SH1247 | 23 | 7 | J | KS | Disturbed |
| SH1247 | 11.8 | 1 | J | KS | Disturbed |
| SH1247 | 15.6 | 2 | J | KS | Disturbed |
| SH1247 | 48.1 | 3 | J | KS | Disturbed |
| SH1247 | 61.2 | 4 | J | KS | Disturbed |
| SH1247 | 52.9 | 5 | J | KS | Disturbed |
| SH1247 | 30.5 | 6 | J | KS | Disturbed |
| SH1247 | 72.8 | 7 | J | KS | Disturbed |
| SH1281 | 18.1 | 1 | J | KS | Agricultural |
| SH1281 | 21.4 | 2 | J | KS | Agricultural |
| SH1281 | 34 | 3 | J | KS | Agricultural |
| SH1281 | 33.3 | 4 | J | KS | Agricultural |
| SH1281 | 30.6 | 5 | J | KS | Agricultural |
| SH1281 | 49.9 | 6 | J | KS | Agricultural |
| SH1281 | 69 | 7 | J | KS | Agricultural |
| SH1281 | 13 | 1 | J | KS | Agricultural |
| SH1281 | 29.6 | 2 | J | KS | Agricultural |
| SH1281 | 17.2 | 3 | J | KS | Agricultural |
| SH1281 | 28 | 4 | J | KS | Agricultural |
| SH1281 | 29 | 5 | J | KS | Agricultural |
| SH1281 | 55 | 6 | J | KS | Agricultural |
| SH1281 | 60.2 | 7 | J | KS | Agricultural |
| SH1281 | 24 | 1 | J | KS | Agricultural |
| SH1281 | 22.9 | 2 | J | KS | Agricultural |
| SH1281 | 24.4 | 3 | J | KS | Agricultural |
| SH1281 | 31.4 | 4 | J | KS | Agricultural |
| SH1281 | 31.4 | 5 | J | KS | Agricultural |
| SH1281 | 19.6 | 6 | J | KS | Agricultural |
| SH1281 | 38 | 7 | J | KS | Agricultural |
| SH1281 | 14.7 | 1 | J | KS | Agricultural |
| SH1281 | 20.1 | 2 | J | KS | Agricultural |
| SH1281 | 19.1 | 3 | J | KS | Agricultural |
| SH1281 | 30.4 | 4 | J | KS | Agricultural |
| SH1281 | 28.4 | 5 | J | KS | Agricultural |
| SH1281 | 61.1 | 6 | J | KS | Agricultural |
| SH1281 | 16.3 | 1 | J | KS | Agricultural |
| SH1281 | 19.6 | 2 | J | KS | Agricultural |
| SH1281 | 14.3 | 3 | J | KS | Agricultural |
| SH1281 | 25.5 | 4 | J | KS | Agricultural |
| SH1281 | 30.3 | 5 | J | KS | Agricultural |
| SH1281 | 51.3 | 6 | J | KS | Agricultural |
| SH1337 | 14.4 | 1 | J | CA | Agricultural |
| SH1337 | 48.4 | 2 | J | CA | Agricultural |
| SH1337 | 29.7 | 3 | J | CA | Agricultural |
| SH1337 | 45.3 | 4 | J | CA | Agricultural |
| SH1337 | 49.5 | 5 | J | CA | Agricultural |
| SH1337 | 72.5 | 6 | J | CA | Agricultural |
| SH1337 | 6 | 1 | J | CA | Agricultural |
| SH1337 | 22.2 | 2 | J | CA | Agricultural |
| SH1337 | 45.7 | 3 | J | CA | Agricultural |
| SH1337 | 45.7 | 4 | J | CA | Agricultural |
| SH1337 | 35.1 | 5 | J | CA | Agricultural |
| SH1337 | 48.35 | 6 | J | CA | Agricultural |
| SH1337 | 65.7 | 7 | J | CA | Agricultural |
| SH1337 | 58.4 | 8 | J | CA | Agricultural |
| SH1337 | 15.6 | 1 | J | CA | Agricultural |
| SH1337 | 42.1 | 2 | J | CA | Agricultural |
| SH1337 | 46.7 | 3 | J | CA | Agricultural |
| SH1337 | 31.2 | 4 | J | CA | Agricultural |
| SH1337 | 19.7 | 5 | J | CA | Agricultural |
| SH1337 | 29.6 | 6 | J | CA | Agricultural |
| SH1337 | 32.6 | 7 | J | CA | Agricultural |
| SH1337 | 40.8 | 8 | J | CA | Agricultural |
| SH1337 | 18.1 | 1 | J | CA | Agricultural |
| SH1337 | 33.6 | 2 | J | CA | Agricultural |
| SH1337 | 37.8 | 3 | J | CA | Agricultural |
| SH1337 | 26.3 | 4 | J | CA | Agricultural |
| SH1337 | 26.3 | 5 | J | CA | Agricultural |
| SH1337 | 29.3 | 6 | J | CA | Agricultural |
| SH1337 | 29.3 | 7 | J | CA | Agricultural |
| SH1337 | 29.3 | 8 | J | CA | Agricultural |
| SH1337 | 10.8 | 1 | J | CA | Agricultural |
| SH1337 | 16.4 | 2 | J | CA | Agricultural |
| SH1337 | 19.4 | 3 | J | CA | Agricultural |
| SH1337 | 23.6 | 4 | J | CA | Agricultural |
| SH1337 | 31.7 | 5 | J | CA | Agricultural |
| SH1337 | 38.5 | 6 | J | CA | Agricultural |
| SH1337 | 77.5 | 7 | J | CA | Agricultural |
| SH1337 | 27 | 8 | J | CA | Agricultural |
| SH1337 | 33.7 | 1 | J | CA | Agricultural |
| SH1337 | 24.2 | 2 | J | CA | Agricultural |
| SH1337 | 50.5 | 3 | J | CA | Agricultural |
| SH1337 | 77.2 | 4 | J | CA | Agricultural |
| SH1337 | 46.1 | 5 | J | CA | Agricultural |
| SH1337 | 51.4 | 6 | J | CA | Agricultural |
| SH1337 | 85.7 | 7 | J | CA | Agricultural |
| SH1337 | 30.4 | 1 | J | CA | Agricultural |
| SH1337 | 32 | 2 | J | CA | Agricultural |
| SH1337 | 40.2 | 3 | J | CA | Agricultural |
| SH1337 | 28.6 | 4 | J | CA | Agricultural |
| SH1337 | 50.2 | 5 | J | CA | Agricultural |
| SH1337 | 37.7 | 6 | J | CA | Agricultural |
| SH1337 | 37.8 | 7 | J | CA | Agricultural |
| SH1337 | 17.8 | 1 | J | CA | Agricultural |
| SH1337 | 27.9 | 2 | J | CA | Agricultural |
| SH1337 | 20.8 | 3 | J | CA | Agricultural |
| SH1337 | 30.4 | 4 | J | CA | Agricultural |
| SH1337 | 49.8 | 5 | J | CA | Agricultural |
| SH1337 | 60.3 | 6 | J | CA | Agricultural |
| SH1337 | 10 | 1 | J | CA | Agricultural |
| SH1337 | 20.8 | 2 | J | CA | Agricultural |
| SH1337 | 15.5 | 3 | J | CA | Agricultural |
| SH1337 | 14.6 | 4 | J | CA | Agricultural |
| SH1337 | 30.2 | 5 | J | CA | Agricultural |
| SH1337 | 50.7 | 6 | J | CA | Agricultural |
| SH1337 | 11 | 1 | J | CA | Agricultural |
| SH1337 | 16.6 | 2 | J | CA | Agricultural |
| SH1337 | 20.2 | 3 | J | CA | Agricultural |
| SH1337 | 17.7 | 4 | J | CA | Agricultural |
| SH1337 | 22 | 5 | J | CA | Agricultural |
| SH1337 | 41.5 | 6 | J | CA | Agricultural |
| SH1337 | 70.4 | 7 | J | CA | Agricultural |
| SH1337 | 17.4 | 1 | J | CA | Agricultural |
| SH1337 | 28 | 2 | J | CA | Agricultural |
| SH1337 | 18.4 | 3 | J | CA | Agricultural |
| SH1337 | 29.1 | 4 | J | CA | Agricultural |
| SH1337 | 42.5 | 5 | J | CA | Agricultural |
| SH1337 | 50.5 | 6 | J | CA | Agricultural |
| SH1337 | 10 | 1 | J | CA | Agricultural |
| SH1337 | 22.2 | 2 | J | CA | Agricultural |
| SH1337 | 26.8 | 3 | J | CA | Agricultural |
| SH1337 | 41.4 | 4 | J | CA | Agricultural |
| SH1337 | 55.4 | 5 | J | CA | Agricultural |
| SH1337 | 46.8 | 6 | J | CA | Agricultural |
| SH1337 | 50.3 | 7 | J | CA | Agricultural |
| SH1350 | 12.7 | 1 | J | CA | Roadside |
| SH1350 | 13.1 | 2 | J | CA | Roadside |
| SH1350 | 13.1 | 3 | J | CA | Roadside |
| SH1350 | 18.3 | 4 | J | CA | Roadside |
| SH1350 | 21.3 | 5 | J | CA | Roadside |
| SH1350 | 23.4 | 6 | J | CA | Roadside |
| SH1350 | 37.2 | 7 | J | CA | Roadside |
| SH1350 | 59.8 | 8 | J | CA | Roadside |
| SH1350 | 13.9 | 1 | J | CA | Roadside |
| SH1350 | 16.2 | 2 | J | CA | Roadside |
| SH1350 | 17.5 | 3 | J | CA | Roadside |
| SH1350 | 24.2 | 4 | J | CA | Roadside |
| SH1350 | 64.2 | 5 | J | CA | Roadside |
| SH1350 | 65.5 | 6 | J | CA | Roadside |
| SH1350 | 14.1 | 1 | J | CA | Roadside |
| SH1350 | 16.1 | 2 | J | CA | Roadside |
| SH1350 | 14.7 | 3 | J | CA | Roadside |
| SH1350 | 14 | 4 | J | CA | Roadside |
| SH1350 | 13.3 | 5 | J | CA | Roadside |
| SH1350 | 24.4 | 6 | J | CA | Roadside |
| SH1350 | 29 | 7 | J | CA | Roadside |
| SH1350 | 46.7 | 8 | J | CA | Roadside |
| SH1350 | 16 | 1 | J | CA | Roadside |
| SH1350 | 19.8 | 2 | J | CA | Roadside |
| SH1350 | 19 | 3 | J | CA | Roadside |
| SH1350 | 30.6 | 4 | J | CA | Roadside |
| SH1350 | 34.9 | 5 | J | CA | Roadside |
| SH1350 | 20.4 | 6 | J | CA | Roadside |
| SH1350 | 20.5 | 7 | J | CA | Roadside |
| SH1350 | 12.3 | 1 | J | CA | Roadside |
| SH1350 | 12.5 | 2 | J | CA | Roadside |
| SH1350 | 16.8 | 3 | J | CA | Roadside |
| SH1350 | 27.7 | 4 | J | CA | Roadside |
| SH1350 | 61.8 | 5 | J | CA | Roadside |
| SH1350 | 57.8 | 6 | J | CA | Roadside |
| SH1350 | 58 | 7 | J | CA | Roadside |
| SH1350 | 12.1 | 1 | J | CA | Roadside |
| SH1350 | 11.9 | 2 | J | CA | Roadside |
| SH1350 | 18.6 | 3 | J | CA | Roadside |
| SH1350 | 48.1 | 4 | J | CA | Roadside |
| SH1350 | 62.6 | 5 | J | CA | Roadside |
| SH1350 | 62.8 | 6 | J | CA | Roadside |
| SH1409 | 14.5 | 1 | J | AZ |  |
| SH1409 | 17.4 | 2 | J | AZ |  |
| SH1409 | 24.4 | 3 | J | AZ |  |
| SH1409 | 32 | 4 | J | AZ |  |
| SH1409 | 40.8 | 5 | J | AZ |  |
| SH1409 | 48.4 | 6 | J | AZ |  |
| SH1409 | 41.1 | 7 | J | AZ |  |
| SH1409 | 64 | 8 | J | AZ |  |
| SH1409 | 18.6 | 1 | J | AZ |  |
| SH1409 | 16.6 | 2 | J | AZ |  |
| SH1409 | 23.5 | 3 | J | AZ |  |
| SH1409 | 28.8 | 4 | J | AZ |  |
| SH1409 | 40.1 | 5 | J | AZ |  |
| SH1409 | 17.6 | 6 | J | AZ |  |
| SH1409 | 38.6 | 7 | J | AZ |  |
| SH1409 | 44.8 | 8 | J | AZ |  |
| SH1409 | 35.6 | 1 | J | AZ |  |
| SH1409 | 30.6 | 2 | J | AZ |  |
| SH1409 | 33 | 3 | J | AZ |  |
| SH1409 | 36.5 | 4 | J | AZ |  |
| SH1409 | 39.2 | 5 | J | AZ |  |
| SH1409 | 16.5 | 6 | J | AZ |  |
| SH1409 | 18.3 | 1 | J | AZ |  |
| SH1409 | 14 | 2 | J | AZ |  |
| SH1409 | 18 | 3 | J | AZ |  |
| SH1409 | 31.5 | 4 | J | AZ |  |
| SH1409 | 32.2 | 5 | J | AZ |  |
| SH1409 | 27.2 | 6 | J | AZ |  |
| SH1409 | 22.4 | 7 | J | AZ |  |
| SH1409 | 53.9 | 8 | J | AZ |  |
| SH1409 | 13.1 | 1 | J | AZ |  |
| SH1409 | 13 | 2 | J | AZ |  |
| SH1409 | 15.2 | 3 | J | AZ |  |
| SH1409 | 22 | 4 | J | AZ |  |
| SH1409 | 48.5 | 5 | J | AZ |  |
| SH1409 | 13.5 | 6 | J | AZ |  |
| SH1409 | 50.6 | 7 | J | AZ |  |
| SH1450 | 21.9 | 1 | J |  |  |
| SH1450 | 54.4 | 2 | J |  |  |
| SH1450 | 54.4 | 3 | J |  |  |
| SH1450 | 45.2 | 4 | J |  |  |
| SH1450 | 56.4 | 5 | J |  |  |
| SH1450 | 21.6 | 1 | J |  |  |
| SH1450 | 43.3 | 2 | J |  |  |
| SH1450 | 49.8 | 3 | J |  |  |
| SH1450 | 64.7 | 4 | J |  |  |
| SH1450 | 69.7 | 5 | J |  |  |
| SH1450 | 20.6 | 1 | J |  |  |
| SH1450 | 49.7 | 2 | J |  |  |
| SH1450 | 49.9 | 3 | J |  |  |
| SH1450 | 62.6 | 4 | J |  |  |
| SH1450 | 55.9 | 5 | J |  |  |
| SH1450 | 52.3 | 6 | J |  |  |
| SH1450 | 17.8 | 1 | J |  |  |
| SH1450 | 44.8 | 2 | J |  |  |
| SH1450 | 70.2 | 3 | J |  |  |
| SH1450 | 57.5 | 4 | J |  |  |
| SH1450 | 73.9 | 5 | J |  |  |
| SH1450 | 23.3 | 1 | J |  |  |
| SH1450 | 41.7 | 2 | J |  |  |
| SH1450 | 67 | 3 | J |  |  |
| SH1450 | 67 | 4 | J |  |  |
| SH1450 | 49.6 | 5 | J |  |  |
| SH1450 | 59.6 | 6 | J |  |  |
| SH1450 | 59.6 | 7 | J |  |  |
| SH1450 | 7 | 1 | J |  |  |
| SH1450 | 13.9 | 2 | J |  |  |
| SH1450 | 16.3 | 3 | J |  |  |
| SH1450 | 16.3 | 4 | J |  |  |
| SH1450 | 14.7 | 5 | J |  |  |
| SH1450 | 29.1 | 6 | J |  |  |
| SH1450 | 89.3 | 7 | J |  |  |
| SH1450 | 89.3 | 8 | J |  |  |
| SH1450 | 30.6 | 1 | J |  |  |
| SH1450 | 33.8 | 2 | J |  |  |
| SH1450 | 40.9 | 3 | J |  |  |
| SH1450 | 64.4 | 4 | J |  |  |
| SH1450 | 60.6 | 5 | J |  |  |
| SH1450 | 64.9 | 6 | J |  |  |
| SH1450 | 9.9 | 1 | J |  |  |
| SH1450 | 19.7 | 2 | J |  |  |
| SH1450 | 27.8 | 3 | J |  |  |
| SH1450 | 33.2 | 4 | J |  |  |
| SH1450 | 34.5 | 5 | J |  |  |
| SH1450 | 61.7 | 6 | J |  |  |
| SH1450 | 37.9 | 7 | J |  |  |
| SH1450 | 70.1 | 8 | J |  |  |
| SH1450 | 8.7 | 1 | J |  |  |
| SH1450 | 17.3 | 2 | J |  |  |
| SH1450 | 22.6 | 3 | J |  |  |
| SH1450 | 28.7 | 4 | J |  |  |
| SH1450 | 30.3 | 5 | J |  |  |
| SH1450 | 51.4 | 6 | J |  |  |
| SH1450 | 37.8 | 7 | J |  |  |
| SH1450 | 67 | 8 | J |  |  |
| SH1450 | 11.3 | 1 | J |  |  |
| SH1450 | 21.4 | 2 | J |  |  |
| SH1450 | 29.3 | 3 | J |  |  |
| SH1450 | 47.7 | 4 | J |  |  |
| SH1450 | 50.1 | 5 | J |  |  |
| SH1450 | 63 | 6 | J |  |  |
| SH1450 | 11 | 1 | J |  |  |
| SH1450 | 13.3 | 2 | J |  |  |
| SH1450 | 28.8 | 3 | J |  |  |
| SH1450 | 21.3 | 4 | J |  |  |
| SH1450 | 49.6 | 5 | J |  |  |
| SH1450 | 53 | 6 | J |  |  |
| SH1450 | 13.1 | 1 | J |  |  |
| SH1450 | 13.9 | 2 | J |  |  |
| SH1450 | 16 | 3 | J |  |  |
| SH1450 | 32.9 | 4 | J |  |  |
| SH1450 | 35.7 | 5 | J |  |  |
| SH1450 | 31.5 | 6 | J |  |  |
| SH1450 | 54.1 | 7 | J |  |  |
| SH1450 | 12.3 | 1 | J |  |  |
| SH1450 | 15 | 2 | J |  |  |
| SH1450 | 20.8 | 3 | J |  |  |
| SH1450 | 38.9 | 4 | J |  |  |
| SH1450 | 37.7 | 5 | J |  |  |
| SH1450 | 48.2 | 6 | J |  |  |
| SH1450 | 12 | 1 | J |  |  |
| SH1450 | 22 | 2 | J |  |  |
| SH1450 | 31 | 3 | J |  |  |
| SH1450 | 46.5 | 4 | J |  |  |
| SH1450 | 53.9 | 5 | J |  |  |
| SH1450 | 77.8 | 6 | J |  |  |
| SH1450 | 41.8 | 7 | J |  |  |
| SH1450 | 9.1 | 1 | J |  |  |
| SH1450 | 11.3 | 2 | J |  |  |
| SH1450 | 16.2 | 3 | J |  |  |
| SH1450 | 28.7 | 4 | J |  |  |
| SH1450 | 27.4 | 5 | J |  |  |
| SH1450 | 50.3 | 6 | J |  |  |
| SH1450 | 76.8 | 7 | J |  |  |
| SH1450 | 66 | 8 | J |  |  |
| SH1450 | 14.4 | 1 | J |  |  |
| SH1450 | 21.1 | 2 | J |  |  |
| SH1450 | 19.5 | 3 | J |  |  |
| SH1450 | 36.7 | 4 | J |  |  |
| SH1450 | 42.7 | 5 | J |  |  |
| SH1450 | 73.7 | 6 | J |  |  |
| SH1450 | 71.5 | 7 | J |  |  |
| SH1450 | 80 | 8 | J |  |  |
| SH1450 | 12 | 1 | J |  |  |
| SH1450 | 15.4 | 2 | J |  |  |
| SH1450 | 27.4 | 3 | J |  |  |
| SH1450 | 33.9 | 4 | J |  |  |
| SH1450 | 35.6 | 5 | J |  |  |
| SH1450 | 35.5 | 6 | J |  |  |
| SH1457 | 11.5 | 1 | J | AZ | Disturbed |
| SH1457 | 13.3 | 2 | J | AZ | Disturbed |
| SH1457 | 13.1 | 3 | J | AZ | Disturbed |
| SH1457 | 18 | 4 | J | AZ | Disturbed |
| SH1457 | 35 | 5 | J | AZ | Disturbed |
| SH1457 | 80.4 | 6 | J | AZ | Disturbed |
| SH1457 | 70.4 | 7 | J | AZ | Disturbed |
| SH1457 | 17.8 | 1 | J | AZ | Disturbed |
| SH1457 | 19.7 | 2 | J | AZ | Disturbed |
| SH1457 | 22.4 | 3 | J | AZ | Disturbed |
| SH1457 | 19.1 | 4 | J | AZ | Disturbed |
| SH1457 | 20.9 | 5 | J | AZ | Disturbed |
| SH1457 | 32.3 | 6 | J | AZ | Disturbed |
| SH1457 | 28.4 | 7 | J | AZ | Disturbed |
| SH1457 | 47.1 | 8 | J | AZ | Disturbed |
| SH1457 | 6.5 | 1 | J | AZ | Disturbed |
| SH1457 | 13.9 | 2 | J | AZ | Disturbed |
| SH1457 | 24.9 | 3 | J | AZ | Disturbed |
| SH1457 | 23.6 | 4 | J | AZ | Disturbed |
| SH1457 | 36 | 5 | J | AZ | Disturbed |
| SH1457 | 41.7 | 6 | J | AZ | Disturbed |
| SH1457 | 52.6 | 7 | J | AZ | Disturbed |
| SH1457 | 55.6 | 8 | J | AZ | Disturbed |
| SH1457 | 51 | 1 | J | AZ | Disturbed |
| SH1457 | 34 | 2 | J | AZ | Disturbed |
| SH1457 | 17.2 | 3 | J | AZ | Disturbed |
| SH1457 | 37.4 | 4 | J | AZ | Disturbed |
| SH1457 | 32.1 | 5 | J | AZ | Disturbed |
| SH1457 | 52.1 | 6 | J | AZ | Disturbed |
| SH1484 | 67.6 | 1 | J | AZ |  |
| SH1484 | 35.3 | 2 | J | AZ |  |
| SH1484 | 23.7 | 3 | J | AZ |  |
| SH1484 | 29.4 | 4 | J | AZ |  |
| SH1484 | 36.9 | 5 | J | AZ |  |
| SH1484 | 58.3 | 6 | J | AZ |  |
| SH1484 | 52.3 | 7 | J | AZ |  |
| SH1484 | 12.9 | 1 | J | AZ |  |
| SH1484 | 14.1 | 2 | J | AZ |  |
| SH1484 | 30.9 | 3 | J | AZ |  |
| SH1484 | 27.6 | 4 | J | AZ |  |
| SH1484 | 45.6 | 5 | J | AZ |  |
| SH1484 | 41.8 | 6 | J | AZ |  |
| SH1484 | 58.3 | 7 | J | AZ |  |
| SH1484 | 48.9 | 8 | J | AZ |  |
| SH1484 | 14.3 | 1 | J | AZ |  |
| SH1484 | 14.2 | 2 | J | AZ |  |
| SH1484 | 16.5 | 3 | J | AZ |  |
| SH1484 | 16.9 | 4 | J | AZ |  |
| SH1484 | 29.8 | 5 | J | AZ |  |
| SH1484 | 47.9 | 6 | J | AZ |  |
| SH1484 | 61.3 | 7 | J | AZ |  |
| SH1484 | 10.2 | 1 | J | AZ |  |
| SH1484 | 11.8 | 2 | J | AZ |  |
| SH1484 | 15.8 | 3 | J | AZ |  |
| SH1484 | 19.3 | 4 | J | AZ |  |
| SH1484 | 30.3 | 5 | J | AZ |  |
| SH1484 | 38.3 | 6 | J | AZ |  |
| SH1484 | 54 | 7 | J | AZ |  |
| SH1493 | 10 | 1 | J | AZ | Agricultural |
| SH1493 | 14.4 | 2 | J | AZ | Agricultural |
| SH1493 | 20.8 | 3 | J | AZ | Agricultural |
| SH1493 | 27.7 | 4 | J | AZ | Agricultural |
| SH1493 | 41.3 | 5 | J | AZ | Agricultural |
| SH1493 | 63.3 | 6 | J | AZ | Agricultural |
| SH1493 | 48.9 | 7 | J | AZ | Agricultural |
| SH1493 | 49 | 8 | J | AZ | Agricultural |
| SH1493 | 10.8 | 1 | J | AZ | Agricultural |
| SH1493 | 13.4 | 2 | J | AZ | Agricultural |
| SH1493 | 15.4 | 3 | J | AZ | Agricultural |
| SH1493 | 32.1 | 4 | J | AZ | Agricultural |
| SH1493 | 47 | 5 | J | AZ | Agricultural |
| SH1493 | 45.7 | 6 | J | AZ | Agricultural |
| SH1493 | 55.4 | 7 | J | AZ | Agricultural |
| SH1493 | 49.3 | 8 | J | AZ | Agricultural |
| SH1493 | 12.1 | 1 | J | AZ | Agricultural |
| SH1493 | 18.6 | 2 | J | AZ | Agricultural |
| SH1493 | 37.6 | 3 | J | AZ | Agricultural |
| SH1493 | 42.5 | 4 | J | AZ | Agricultural |
| SH1493 | 59.2 | 5 | J | AZ | Agricultural |
| SH1493 | 50.3 | 6 | J | AZ | Agricultural |
| Theis | 23.4 | 1 | S |  |  |
| Theis | 29 | 2 | S |  |  |
| Theis | 44.8 | 3 | S |  |  |
| Theis | 44.8 | 4 | S |  |  |
| Theis | 26.4 | 5 | S |  |  |
| Theis | 27.2 | 6 | S |  |  |
| Theis | 13.4 | 7 | S |  |  |
| Theis | 14.3 | 8 | S |  |  |
| Theis | 38.3 | 1 | S |  |  |
| Theis | 76.4 | 2 | S |  |  |
| Theis | 43.1 | 3 | S |  |  |
| Theis | 37.1 | 4 | S |  |  |
| Theis | 31.5 | 5 | S |  |  |
| Theis | 27.3 | 6 | S |  |  |
| Theis | 20 | 7 | S |  |  |
| Theis | 20 | 8 | S |  |  |
| Theis | 19.4 | 1 | S |  |  |
| Theis | 20.5 | 2 | S |  |  |
| Theis | 19.1 | 3 | S |  |  |
| Theis | 20.2 | 4 | S |  |  |
| Theis | 20.3 | 5 | S |  |  |
| Theis | 33.5 | 6 | S |  |  |
| Theis | 33.8 | 7 | S |  |  |
| Theis | 37.8 | 8 | S |  |  |
| Theis | 21.7 | 1 | S |  |  |
| Theis | 19.3 | 2 | S |  |  |
| Theis | 24.6 | 3 | S |  |  |
| Theis | 25.9 | 4 | S |  |  |
| Theis | 45.3 | 5 | S |  |  |
| Theis | 29.8 | 6 | S |  |  |
| Theis | 27 | 7 | S |  |  |
| Theis | 16 | 1 | S |  |  |
| Theis | 25.5 | 2 | S |  |  |
| Theis | 24.3 | 3 | S |  |  |
| Theis | 18.4 | 4 | S |  |  |
| Theis | 17 | 5 | S |  |  |
| Theis | 24.8 | 6 | S |  |  |
| Theis | 35.1 | 7 | S |  |  |
| Theis | 15.6 | 1 | S |  |  |
| Theis | 19.2 | 2 | S |  |  |
| Theis | 27.8 | 3 | S |  |  |
| Theis | 33.1 | 4 | S |  |  |
| Theis | 91 | 5 | S |  |  |
| Theis | 35.4 | 6 | S |  |  |
| Theis | 52.8 | 7 | S |  |  |
| Theis | 48 | 8 | S |  |  |
