# Supplementary material for: Leaf angle distribution in Johnsongrass, leaf thickness in sorghum and Johnsongrass, and association with response to Colletotrichum sublineola
Source: Sci Rep. 2020 Dec 18;10:22320. doi: 10.1038/s41598-020-79473-x (PMC7749152; doi:10.1038/s41598-020-79473-x)
Supplement: Supplementary file 2 — Supplementary Table S2. [file 41598_2020_79473_MOESM2_ESM.docx]

Title of the manuscript: Leaf angle distribution in Johnsongrass, leaf thickness in sorghum and Johnsongrass, and association with response to *Colletotrichum sublineola*

Full author list: EZEKIEL AHN^1^, GARY ODVODY^2^, LOUIS K. PROM^3^ and CLINT MAGILL^1^

^1^*Department of Plant Pathology & Microbiology, Texas A&M University, College Station, TX, USA*

^2^ *Texas A&M AgriLife Research, Corpus Christi, TX, USA*

^3^ *USDA-ARS Southern Plains Agricultural Research Center, College Station, TX, USA*

Correspondence to: C. Magill. E-mail: c-magill@tamu.edu

Supplementary Table S2. Raw data of leaf thickness and corresponding susceptibility to *C. sublineola*. Name of plant cultivars, leaf blade and midrib thickness at apex, at base, and at half-way between apex and base are listed. Corresponding susceptibilities at the points are listed (LB=leaf blade, ML=midrib). Four top leaves were used. Different replications were separated by space.

| Theis | Apex LB (mm) | Susceptibility | Apex ML  (mm) | Susceptibility | Middle LB (mm) | Susceptibility |
| --- | --- | --- | --- | --- | --- | --- |
| Leaf 1 | 0.2 | 10 | 0.2 | 10 | 0.14 | 10 |
| Leaf 2 | 0.14 | 10 | 0.14 | 10 | 0.15 | 1 |
| Leaf 3 | 0.19 | 10 | 0.19 | 10 | 0.15 | 6 |
| Leaf 4 | 0.15 | 10 | 0.15 | 10 | 0.17 | 10 |
|  |  |  |  |  |  |  |
| Leaf 1 | 0.22 | 10 | 0.22 | 10 | 0.18 | 10 |
| Leaf 2 | 0.16 | 10 | 0.16 | 10 | 0.18 | 4 |
| Leaf 3 | 0.21 | 10 | 0.21 | 10 | 0.18 | 10 |
| Leaf 4 | 0.16 | 10 | 0.16 | 10 | 0.18 | 10 |
|  |  |  |  |  |  |  |
| Leaf 1 | 0.2 | 10 | 0.2 | 10 | 0.15 | 1 |
| Leaf 2 | 0.13 | 10 | 0.13 | 10 | 0.15 | 10 |
| Leaf 3 | 0.22 | 10 | 0.22 | 10 | 0.16 | 10 |
| Leaf 4 | 0.35 | 10 | 0.35 | 10 | 0.15 | 10 |
|  |  |  |  |  |  |  |
| Leaf 1 | 0.14 | 1 | 0.14 | 1 | 0.14 | 10 |
| Leaf 2 | 0.14 | 8 | 0.14 | 8 | 0.15 | 1 |
| Leaf 3 | 0.19 | 10 | 0.19 | 10 | 0.16 | 1 |
| Leaf 4 | 0.23 | 10 | 0.23 | 10 | 0.16 | 10 |
|  |  |  |  |  |  |  |
| Theis | Middle ML (mm) | Susceptibility | Base LB  (mm) | Susceptibility | Base ML  (mm) | Susceptibility  (mm) |
| Leaf 1 | 0.34 | 1 | 0.13 | 10 | 0.58 | 10 |
| Leaf 2 | 0.75 | 1 | 0.15 | 10 | 1.37 | 1 |
| Leaf 3 | 0.95 | 1 | 0.18 | 9 | 1.93 | 1 |
| Leaf 4 | 1.22 | 6 | 0.22 | 5 | 1.95 | 1 |
|  |  |  |  |  |  |  |
| Leaf 1 | 0.32 | 1 | 0.24 | 10 | 0.88 | 1 |
| Leaf 2 | 0.7 | 1 | 0.28 | 1 | 1.63 | 1 |
| Leaf 3 | 0.71 | 1 | 0.18 | 1 | 1.79 | 1 |
| Leaf 4 | 1.26 | 5 | 0.21 | 10 | 2.71 | 1 |
|  |  |  |  |  |  |  |
| Leaf 1 | 0.42 | 1 | 0.18 | 10 | 0.91 | 1 |
| Leaf 2 | 0.63 | 1 | 0.19 | 10 | 1.89 | 5 |
| Leaf 3 | 0.86 | 3 | 0.16 | 10 | 2.16 | 1 |
| Leaf 4 | 1.42 | 3 | 0.22 | 10 | 2.89 | 5 |
|  |  |  |  |  |  |  |
| Leaf 1 | 0.33 | 1 | 0.17 | 10 | 0.67 | 1 |
| Leaf 2 | 0.65 | 1 | 0.21 | 8 | 1.75 | 1 |
| Leaf 3 | 0.71 | 1 | 0.16 | 1 | 1.78 | 1 |
| Leaf 4 | 1.17 | 1 | 0.26 | 10 | 2.35 | 1 |
|  |  |  |  |  |  |  |
| QL3 | Apex LB  (mm) | Susceptibility | Apex ML  (mm) | Susceptibility | Middle LB  (mm) | Susceptibility |
| Leaf 1 | 0.15 | 1 | 0.15 | 1 | 0.16 | 10 |
| Leaf 2 | 0.19 | 10 | 0.19 | 10 | 0.18 | 10 |
| Leaf 3 | 0.2 | 10 | 0.2 | 10 | 0.15 | 10 |
| Leaf 4 | 0.17 | 10 | 0.17 | 10 | 0.16 | 10 |
|  |  |  |  |  |  |  |
| Leaf 1 | 0.22 | 10 | 0.22 | 10 | 0.18 | 10 |
| Leaf 2 | 0.21 | 10 | 0.21 | 10 | 0.16 | 10 |
| Leaf 3 | 0.21 | 10 | 0.21 | 10 | 0.17 | 10 |
| Leaf 4 | 0.18 | 10 | 0.18 | 10 | 0.16 | 10 |
|  |  |  |  |  |  |  |
| Leaf 1 | 0.21 | 10 | 0.21 | 10 | 0.15 | 10 |
| Leaf 2 | 0.22 | 10 | 0.22 | 10 | 0.16 | 1 |
| Leaf 3 | 0.19 | 10 | 0.19 | 10 | 0.16 | 5 |
| Leaf 4 | 0.17 | 10 | 0.17 | 10 | 0.19 | 10 |
|  |  |  |  |  |  |  |
| Leaf 1 | 0.21 | 10 | 0.21 | 10 | 0.15 | 10 |
| Leaf 2 | 0.27 | 10 | 0.27 | 10 | 0.16 | 8 |
| Leaf 3 | 0.2 | 10 | 0.2 | 10 | 0.18 | 10 |
| Leaf 4 | 0.18 | 10 | 0.18 | 10 | 0.14 | 10 |
|  |  |  |  |  |  |  |
| Leaf 1 | 0.18 | 10 | 0.18 | 10 | 0.16 | 10 |
| Leaf 2 | 0.2 | 10 | 0.2 | 10 | 0.16 | 4 |
| Leaf 3 | 0.18 | 10 | 0.18 | 10 | 0.16 | 10 |
| Leaf 4 | 0.22 | 10 | 0.22 | 10 | 0.2 | 10 |
|  |  |  |  |  |  |  |
| QL3 | Middle ML  (mm) | Susceptibility | Base LB  (mm) | Susceptibility | Base ML  (mm) | Susceptibility |
| Leaf 1 | 0.6 | 1 | 0.16 | 10 | 0.95 | 1 |
| Leaf 2 | 0.94 | 1 | 0.17 | 10 | 2.16 | 1 |
| Leaf 3 | 0.81 | 1 | 0.17 | 10 | 2.25 | 1 |
| Leaf 4 | 1.01 | 2 | 0.15 | 10 | 2.36 | 2 |
|  |  |  |  |  |  |  |
| Leaf 1 | 0.26 | 1 | 0.17 | 10 | 0.45 | 1 |
| Leaf 2 | 0.6 | 1 | 0.17 | 10 | 1.12 | 1 |
| Leaf 3 | 0.91 | 1 | 0.2 | 1 | 2.19 | 1 |
| Leaf 4 | 0.81 | 1 | 0.24 | 10 | 2.38 | 1 |
|  |  |  |  |  |  |  |
| Leaf 1 | 0.47 | 8 | 0.15 | 10 | 0.81 | 1 |
| Leaf 2 | 0.85 | 1 | 0.17 | 10 | 1.93 | 1 |
| Leaf 3 | 1 | 1 | 0.17 | 3 | 2.39 | 1 |
| Leaf 4 | 1.13 | 1 | 0.22 | 10 | 2.73 | 1 |
|  |  |  |  |  |  |  |
| Leaf 1 | 0.39 | 1 | 0.16 | 10 | 0.67 | 1 |
| Leaf 2 | 0.73 | 1 | 0.17 | 10 | 1.96 | 1 |
| Leaf 3 | 0.75 | 1 | 0.31 | 1 | 2.12 | 1 |
| Leaf 4 | 0.98 | 1 | 0.28 | 10 | 2.66 | 1 |
|  |  |  |  |  |  |  |
| Leaf 1 | 0.5 | 1 | 0.17 | 10 | 1.03 | 1 |
| Leaf 2 | 0.92 | 1 | 0.22 | 10 | 2.3 | 1 |
| Leaf 3 | 0.75 | 1 | 0.19 | 10 | 1.84 | 1 |
| Leaf 4 | 1.22 | 1 | 0.24 | 10 | 2.33 | 1 |
|  |  |  |  |  |  |  |
| RTx2536 | Apex LB  (mm) | Susceptibility | Apex ML  (mm) | Susceptibility | Middle LB  (mm) | Susceptibility |
| Leaf 1 | 0.27 | 10 | 0.27 | 10 | 0.18 | 10 |
| Leaf 2 | 0.21 | 10 | 0.21 | 10 | 0.19 | 10 |
| Leaf 3 | 0.22 | 10 | 0.22 | 10 | 0.21 | 10 |
| Leaf 4 | 0.18 | 10 | 0.18 | 10 | 0.2 | 10 |
|  |  |  |  |  |  |  |
| Leaf 1 | 0.3 | 10 | 0.3 | 10 | 0.18 | 10 |
| Leaf 2 | 0.3 | 10 | 0.3 | 10 | 0.17 | 10 |
| Leaf 3 | 0.24 | 10 | 0.24 | 10 | 0.19 | 10 |
| Leaf 4 | 0.2 | 4 | 0.2 | 4 | 0.18 | 10 |
|  |  |  |  |  |  |  |
| Leaf 1 | 0.24 | 10 | 0.24 | 10 | 0.17 | 10 |
| Leaf 2 | 0.22 | 10 | 0.22 | 10 | 0.19 | 10 |
| Leaf 3 | 0.19 | 10 | 0.19 | 10 | 0.18 | 5 |
| Leaf 4 | 0.23 | 10 | 0.23 | 10 | 0.18 | 5 |
|  |  |  |  |  |  |  |
| RTx2536 | Middle ML  (mm) | Susceptibility | Base LB  (mm) | Susceptibility | Base ML  (mm) | Susceptibility |
| Leaf 1 | 0.42 | 2 | 0.2 | 10 | 1.56 | 1 |
| Leaf 2 | 1.08 | 1 | 0.19 | 10 | 2.82 | 4 |
| Leaf 3 | 1.46 | 1 | 0.21 | 10 | 3.78 | 10 |
| Leaf 4 | 1.57 | 1 | 0.19 | 10 | 3.21 | 1 |
|  |  |  |  |  |  |  |
| Leaf 1 | 0.5 | 1 | 0.24 | 10 | 1.44 | 1 |
| Leaf 2 | 0.91 | 1 | 0.22 | 10 | 2.21 | 1 |
| Leaf 3 | 1.27 | 1 | 0.16 | 10 | 2.59 | 1 |
| Leaf 4 | 1.45 | 1 | 0.23 | 10 | 2.94 | 1 |
|  |  |  |  |  |  |  |
| Leaf 1 | 0.44 | 1 | 0.2 | 10 | 1.15 | 1 |
| Leaf 2 | 1.2 | 1 | 0.22 | 10 | 2.63 | 1 |
| Leaf 3 | 1.3 | 2 | 0.22 | 7 | 3.18 | 2 |
| Leaf 4 | 1.16 | 3 | 0.22 | 5 | 2.82 | 1 |
|  |  |  |  |  |  |  |
| SH1247 | Apex LB  (mm) | Susceptibility | Apex ML  (mm) | Susceptibility | Middle LB  (mm) | Susceptibility |
| Leaf 1 | 0.27 | 10 | 0.27 | 10 | 0.32 | 10 |
| Leaf 2 | 0.23 | 10 | 0.23 | 10 | 0.19 | 10 |
| Leaf 3 | 0.32 | 10 | 0.32 | 10 | 0.17 | 10 |
| Leaf 4 | 0.19 | 10 | 0.19 | 10 | 0.19 | 10 |
|  |  |  |  |  |  |  |
| Leaf 1 | 0.21 | 10 | 0.21 | 10 | 0.16 | 10 |
| Leaf 2 | 0.25 | 10 | 0.25 | 10 | 0.23 | 7 |
| Leaf 3 | 0.22 | 10 | 0.22 | 10 | 0.19 | 10 |
| Leaf 4 | 0.22 | 10 | 0.22 | 10 | 0.16 | 10 |
|  |  |  |  |  |  |  |
| Leaf 1 | 0.24 | 10 | 0.24 | 10 | 0.24 | 10 |
| Leaf 2 | 0.2 | 10 | 0.2 | 10 | 0.18 | 10 |
| Leaf 3 | 0.2 | 10 | 0.2 | 10 | 0.15 | 10 |
| Leaf 4 | 0.21 | 10 | 0.21 | 10 | 0.15 | 10 |
|  |  |  |  |  |  |  |
| Leaf 1 | 0.27 | 10 | 0.27 | 10 | 0.18 | 10 |
| Leaf 2 | 0.21 | 10 | 0.21 | 10 | 0.2 | 10 |
| Leaf 3 | 0.23 | 10 | 0.23 | 10 | 0.18 | 10 |
| Leaf 4 | 0.27 | 10 | 0.27 | 10 | 0.17 | 10 |
|  |  |  |  |  |  |  |
| Leaf 1 | 0.27 | 10 | 0.27 | 10 | 0.27 | 10 |
| Leaf 2 | 0.23 | 10 | 0.23 | 10 | 0.17 | 10 |
| Leaf 3 | 0.24 | 10 | 0.24 | 10 | 0.15 | 10 |
| Leaf 4 | 0.2 | 10 | 0.2 | 10 | 0.18 | 10 |
|  |  |  |  |  |  |  |
| SH1247 | Middle ML  (mm) | Susceptibility | Base LB  (mm) | Susceptibility | Base ML  (mm) | Susceptibility |
| Leaf 1 | 0.5 | 3 | 0.22 | 5 | 1.16 | 1 |
| Leaf 2 | 0.93 | 7 | 0.18 | 10 | 2.77 | 1 |
| Leaf 3 | 0.96 | 6 | 0.28 | 10 | 2.64 | 1 |
| Leaf 4 | 1.13 | 8 | 0.18 | 10 | 2.98 | 1 |
|  |  |  |  |  |  |  |
| Leaf 1 | 0.73 | 2 | 0.26 | 10 | 1.94 | 1 |
| Leaf 2 | 0.54 | 1 | 0.29 | 2 | 1.64 | 1 |
| Leaf 3 | 0.8 | 1 | 0.24 | 3 | 2.36 | 1 |
| Leaf 4 | 0.67 | 5 | 0.17 | 8 | 1.64 | 1 |
|  |  |  |  |  |  |  |
| Leaf 1 | 0.59 | 1 | 0.17 | 10 | 1.3 | 1 |
| Leaf 2 | 0.69 | 1 | 0.16 | 10 | 2.33 | 1 |
| Leaf 3 | 0.73 | 1 | 0.19 | 10 | 1.8 | 1 |
| Leaf 4 | 0.53 | 9 | 0.23 | 5 | 1.47 | 1 |
|  |  |  |  |  |  |  |
| Leaf 1 | 0.47 | 1 | 0.19 | 10 | 1.2 | 1 |
| Leaf 2 | 0.64 | 1 | 0.18 | 10 | 1.98 | 1 |
| Leaf 3 | 0.85 | 6 | 0.2 | 1 | 2.43 | 1 |
| Leaf 4 | 0.78 | 10 | 0.18 | 5 | 2.53 | 1 |
|  |  |  |  |  |  |  |
| Leaf 1 | 0.4 | 10 | 0.19 | 10 | 0.74 | 1 |
| Leaf 2 | 0.53 | 1 | 0.22 | 4 | 1.4 | 2 |
| Leaf 3 | 0.56 | 10 | 0.18 | 6 | 1.47 | 1 |
| Leaf 4 | 0.79 | 10 | 0.17 | 10 | 1.83 | 10 |
